# The lay of the land: Associations between environmental features and personality

**DOI:** 10.1111/jopy.12822

**Published:** 2023-04-08

**Authors:** Ioana E. Militaru, Gregory Serapio‐García, Tobias Ebert, Wenyuan Kong, Samuel D. Gosling, Jeff Potter, Peter J. Rentfrow, Friedrich M. Götz

**Affiliations:** ^1^ Department of Psychology University of Cambridge Cambridge UK; ^2^ Department of Psychology University of Mannheim Mannheim Germany; ^3^ School of Earth and Space Sciences Peking University Beijing China; ^4^ Department of Psychology University of Texas at Austin Austin Texas USA; ^5^ School of Psychological Sciences University of Melbourne Melbourne Australia; ^6^ Atof Inc. Cambridge Massachusetts USA; ^7^ Department of Psychology University of British Columbia Vancouver British Columbia Canada

**Keywords:** Big Five, ecological psychology, geographical psychology, landscape, personality, physical environments

## Abstract

**Objective:**

Personality traits cluster across countries, regions, cities, and neighborhoods. What drives the formation of these clusters? Ecological theory suggests that physical locations shape humans' patterns of behaviors and psychological characteristics. Based on this theory, we examined whether and how differential land‐cover relates to individual personality.

**Method:**

We followed a preregistered three‐pronged analysis approach to investigate the associations between personality (*N* = 2,690,878) and land‐cover across the United States. We used eleven land‐cover categories to classify landscapes and tested their association with personality against broad physical and socioeconomic factors.

**Results:**

Urban areas were positively associated with openness to experience and negatively associated with conscientiousness. Coastal areas were positively associated with openness to experience and neuroticism but negatively associated with agreeableness and conscientiousness. Cultivated areas were negatively associated with openness. Landscapes at the periphery of human activity, such as shrubs, bare lands, or permanent snows, were not reliably associated with personality traits.

**Conclusions:**

Bivariate correlations, multilevel, and random forest models uncovered robust associations between landscapes and personality traits. These findings align with ecological theory suggesting that an individual's environment contributes to their behaviors, thoughts, and feelings.

## 1 INTRODUCTION

Ample evidence from geographical psychology documents personality variation across macroenvironments, such as countries (e.g., McCrae & Terracciano, 2005; Schmitt et al., 2007), administrative areas (e.g., Allik et al., 2009; Ebert et al., 2021), cities (e.g., Bleidorn et al., 2016; Wei et al., 2017), and neighborhoods (e.g., Jokela, 2020; Jokela et al., 2015). What drives the formation of these patterns? Prior theory has posited three potential drivers of geographic variation in personality: selective migration, social influence, and environmental influence (Rentfrow et al., 2008). First, the selective migration hypothesis proposes that individuals are more likely to selectively move to regions that satisfy their needs (e.g., Jokela, 2020; Rentfrow, 2010); in time, this process contributes toward regional clusters of personality traits. Second, local social influences may lead residents to adopt particular traits, values, and behaviors that reinforce the prevailing social norms (e.g., Gelfand et al., 2011; Harrington & Gelfand, 2014). Third, features of the physical environment may create behavioral opportunities and barriers that, over time, influence personality development (Meagher, 2020; Oishi, 2014; Rentfrow et al., 2015; Schaller & Murray, 2008). In the current article, we develop and test the hypothesis that landscapes represent one class of environmental features that relate to spatial variation in personality.

In recent years, an emerging stream of research has identified elements of the physical environment, including climate (e.g., Wei et al., 2017), terrain (Kitayama et al., 2006), and topography (e.g., Götz et al., 2020), that are associated with geographical variation in personality. For example, researchers have hypothesized that individuals growing up in regions with temperatures closer to 22°C experience greater opportunities to explore their environments and socialize than do individuals living in more extreme climates (Van de Vliert, 2013; Van de Vliert et al., 2013; Wei et al., 2017). Compared with hot or cold climates, living in more temperate climates is associated with increased socializing, because such climates afford more opportunities for individuals to explore and interact. These affordances appear to contribute to higher levels of openness, conscientiousness, extraversion, agreeableness, and emotional stability among residents of temperate regions (Wei et al., 2017). Additionally, subsistence styles are dependent on the layout and affordances of agricultural terrain and can, in turn, have important cognitive implications. For example, subsistence styles that require cooperation, such as farming and fishing, are associated with greater interdependent thinking (Uskul et al., 2008). Following this idea, research has shown that rice farming has shaped collectivistic social practices throughout China (Talhelm et al., 2014). Topography, an environmental factor pertaining to the arrangement of a landscape, has also been associated with different patterns of personality traits. For instance, the harsh conditions imposed by mountainous terrain have been hypothesized to contribute to regional differences in independence, individuality, and resourcefulness, which are reflected in higher levels of openness and lower levels of agreeableness, extraversion, neuroticism, and conscientiousness (Götz et al., 2020).

### 1.1 Current research

Building on these findings, we argue that the composition of a landscape (e.g., whether it consists primarily of forests, grasslands, wetlands, or artificial surfaces) may similarly contribute to spatial variation in personality. In the current study, we aim to assess whether there are any meaningful associations between landscapes and personality traits. To this end, we employed multiple preregistered analytical strategies and accounted for a wide range of control variables to evaluate the robustness of our findings (https://osf.io/8j3en/).

Past efforts to investigate the associations between broad environmental factors and personality have focused on countries (Allik & McCrae, 2004; McCrae et al., 2007) and ZIP codes (e.g., Götz et al., 2020; Wei et al., 2017) as geographical units of investigation. The pressures or affordances exerted by environmental factors upon individuals' behaviors likely unfold first and foremost in more immediate, granular environments, such as one's city or neighborhood, and less at the country level, making ZIP codes a more suitable level of analysis (Elleman et al., 2020). Here, we used personality data collected from 3,838,112 U.S. participants. To operationalize the U.S. landscape comprehensively, we retrieved satellite land‐cover data corresponding to 32,657 U.S. ZIP codes. The land‐cover taxonomy, which is used in geographical sciences research (e.g., Anderson, 1976; Chen & Chen, 2018), comprises 11 categories: (1) cultivated land, (2) forest, (3) grassland, (4) shrub, (5) wetland, (6) water bodies, (7) tundra, (8) artificial surface, (9) bare land, (10) glaciers and permanent snow, (11) ocean.

We implemented a three‐stage analytical strategy. First, we examined the zero‐order correlations between land‐cover categories and residents' personality traits. Second, we nested participants into ZIP codes^1^ and ran multilevel analyses to assess the magnitude of the associations between the land‐cover categories and residents' personality traits. In line with prior research (e.g., Ebert et al., 2021; Götz et al., 2020; Jokela et al., 2015; Rentfrow et al., 2013; Wei et al., 2017), we probed the robustness of these associations by controlling for ecological and demographic indicators at the individual (i.e., age, gender, race, education) and ZIP code‐levels (i.e., climate, age, race, education, income). Third, we conducted a series of data‐driven machine‐learning analyses to estimate the relative importance of the predictors for explaining personality traits.

Overall, the current study seeks to offer a granular understanding of the links between personality traits and a suite of physical environmental factors. By investigating the associations between psychological constructs and features of the physical environment, we seek to gain broader knowledge of the impact of the environment on personality. Geographical psychology and socioecological psychology offer overlapping perspectives for understanding the nature of these associations. From a geographical psychology perspective (Rentfrow et al., 2008), associations between land‐cover and personality can inform our understanding of the spatial distribution of personality by offering insights into why certain traits are concentrated in particular geographical areas. From a socioecological psychology perspective (Oishi, 2014), such research can provide deeper understanding of how objective landscapes may affect individuals' subjective experience, which in turn becomes expressed through personality.

### 1.2 Personality and landscape

Previous research has uncovered a positive association between openness to experience and measures of urbanity, such as population density, percentage of foreign‐born residents (Ebert et al., 2021; Rentfrow et al., 2015), and urban amenities (Götz et al., 2021). It is plausible that residents of urban environments regularly engage in new experiences and have a diverse range of social interactions, which might foster characteristics linked to openness, such as curiosity, creativity, and open‐mindedness. Direct evidence supports this hypothesis. People higher in openness live in more urban areas (Jokela et al., 2015), have a preference for urban, cosmopolitan areas (Sevincer et al., 2017), and are more likely to move there (Jokela, 2020; Yoshino & Oshio, 2022). As such, we expected the proportion of artificial surface, which can be regarded as a measure of urbanity, to be positively related to residents' openness (H1a).

We expected land‐cover categories capturing water (i.e., water bodies, wetland, ocean) to be more inviting to exploration and hence positively related to openness to experience (H1b, H1c, H1d). This assumption is supported by the geographical distribution of openness to experience uncovered by Rentfrow et al. (2008), which shows that U.S. coastal states tend to be higher in openness to experience than inland states. Similarly, when investigating self‐reported space preferences, Oishi and Choi (2020) found that openness to experience was positively correlated with a preference for environments that are “oceanlike.”

Urban areas have also been found to be more extraverted than rural areas, perhaps because highly sociable individuals are more likely to move to urban areas and move across greater distances (Jokela et al., 2008). For instance, there is evidence that residents of urban neighborhoods in central London are more extraverted compared to residents living in the outer boroughs (Jokela et al., 2015). As such, we hypothesized that extraversion, which captures individuals' predisposition toward being sociable and assertive (John & Srivastava, 1999), would be positively associated with the proportion of artificial surface (H2a).

Furthermore, building on previous literature, we predicted that extraversion would be positively associated with water bodies (H2b), wetland (H2c), and ocean (H2d). Across multiple studies investigating geographical preferences, extraversion has been associated with a preference for oceans. Specifically, Oishi et al. (2015) found that extraverted participants were more likely to choose places close to oceans when they wanted to socialize.

Lastly, due to the scarcity of prior research to inform our hypotheses, we adopted a preregistered exploratory approach to investigate the relationship between personality traits and the remaining land‐cover types (i.e., grassland, shrub, bare land, glaciers, and permanent snow; https://osf.io/8j3en/).

### 1.3 Landscape versus other broad factors

Are the associations between landscape types and personality independent of other broad factors, such as climate (Wei et al., 2017), gender ratio (Griskevicius et al., 2012), or income (He et al., 2017)? To rigorously address the primary aim of our study, we tested the robustness of the relationship between landscape and personality while controlling for climate and relevant sociodemographic variables. In keeping with previous research investigating the associations between physical environment and personality (Götz et al., 2020), we controlled for ambient economic indicators (median household income and education) and several personal and ambient demographic variables (age, race, gender). We additionally controlled for several ambient climate indicators. Landscape and climate are comparable and interrelated broad environmental factors as change in one tends to induce change in the other (e.g., Kalnay & Cai, 2003; Vose et al., 2004), making climate an important covariate. To this end, we retrieved 30‐year averages of three climatic indicators: temperature, precipitation, and snowfall (National Centers for Environmental Information, 2020, 1991–2020 U.S. Climate Normals) and empirically tested the predictive power of landscapes for individual personality against them.

## 2 METHOD

### 2.1 Participants

Participants were recruited through the Gosling‐Potter Internet Personality Project (GPIPP) between 2002 and 2015 using a non‐commercial, advertisement‐free website (https://www.outofservice.com), which offers users feedback on several measures, including personality inventories.

The research project was approved by the institutional ethics committees of the University of California and the University of Texas (including a waiver of parental consent; for details, see Soto et al., 2008). Since the onset of data collection, the GPIPP dataset has been used in a number of studies in the psychological literature (e.g., Bleidorn et al., 2016; Laajaj et al., 2019; Rentfrow et al., 2013), but it has never been linked with measures of land‐cover. The dataset contains self‐reported sociodemographic information, including gender, ethnicity, age, education, the Big Five personality traits, and participants' current place of residence in the form of ZIP code (*N =* 3,052,333). In line with previous research using the GPIPP dataset (Götz et al., 2020), we excluded participants who reported living outside the United States and who fell outside the 10–99 age range. Further, we implemented a listwise deletion approach and excluded all participants who reported unidentifiable ZIP codes or had missing responses. An inspection of the missing data revealed that social class, one of the participant‐level (i.e., Level 1) predictors, had missing data for 1,271,754 participants from the remaining sample, potentially indicating the sensitive nature of this variable. To maximize statistical power, we deviated from our preregistered strategy and conducted the main analyses without social class, but we included the variable in robustness checks (Supplementary Information, Tables S1–S7). We applied listwise deletion on the remaining variables. Applying all the aforementioned exclusion criteria resulted in a final sample of 2,690,878, where 64.7% self‐reported being female, with an average age of 26.4 (range = [10–99], SD = 11.8). 73.99% self‐identified as White, 9.59% self‐identified as Black, 8.05% as Hispanic, 2.53% as Asian, 1.01% as Mixed, and 4.83% as Other. These gender and racial compositions are typical for online studies (Gosling et al., 2004).

### 2.2 Measures

#### 2.2.1 Personality

Personality was self‐reported using the Big Five Inventory (BFI; John & Srivastava, 1999). Five‐point agreement ratings (ranging from 1 = “Disagree strongly” to 5 = “Agree strongly”) on 44 items assessing each of the Big Five personality traits (openness to experience, conscientiousness, extraversion, agreeableness, and neuroticism) were averaged (after rescoring reverse‐keyed items) to obtain a mean score for each trait.

#### 2.2.2 Land‐cover

Land‐cover composition of U.S. ZIP codes was generated using a predefined taxonomy and was collected from the National Catalogue Service for Geographic Information (https://www.webmap.cn). This approach has been used previously in the geographical literature (e.g., Anderson, 1976; Chen & Chen, 2018) and leverages satellite images to calculate the proportions of the following land‐cover categories: grassland, cultivated land, forest, wetland, water bodies, artificial surface, bare land, glaciers and permanent snow, oceans, and tundra. The data collection was carried out between 2018–2019, while the satellite images were captured in 2010.

In its raw format, the dataset contained pixels classified into one of the 11 land‐cover categories, along with their geolocation, which facilitated mapping onto U.S. ZIP codes (Supplementary Information, Table S1). We eliminated tundra from all subsequent analyses because this category had zero variance, signaling that it is not a represented land‐cover category in the U.S. imagery dataset, as Alaska is not captured in the dataset.

To address the statistical dependence of the ten land‐cover categories (which capture relative proportions and add to 1 within each ZIP code^2^) and in line with our preregistered strategy (https://osf.io/8j3en/), we eliminated forest (which is the category with the highest prevalence across the studied ZIP codes) from subsequent multilevel analyses, hence treating it as a reference category (Supplementary Information, Table S2).

It is important to note that the land‐cover method captured only inland variation. As such, the oceans category represents ocean pixels that were within the boundaries of a ZIP code, such as oceans surrounding peninsulas or islands, and failed to capture ZIP codes that simply border oceans. To address this limitation, we went beyond our preregistered approach and collected additional data from the National Oceanic and Atmospheric Administration (NOAA; Office for Coastal Management, 2022) which included a binary list of coastal and non‐coastal U.S. ZIP codes. We then conducted a series of additional multilevel analyses where we replaced the oceans variable with the coastal measure (Supplementary Information, Tables S3–S7).

#### 2.2.3 Climate

We extracted three climate indices from the National Oceanic and Atmospheric Administration (NOAA; https://www.ncei.noaa.gov)—temperature, snowfall, and precipitation—based on 30‐year historical normal values from almost 15,000 U.S. meteorological stations (James & Arguez, 2015). We retrieved the ZIP codes' centroid latitude and longitude coordinates from the 2016 TIGER Census Bureau's demographic data (United States Census Bureau, 2010) and calculated the mean climate indices retrieved from the closest three weather stations to each centroid.

#### 2.2.4 Demographic variables

In keeping with previous geographical psychology studies (Ebert et al., 2021; Götz et al., 2020; Jokela et al., 2015; Rentfrow et al., 2013), at participant‐level (Level 1) we controlled for gender, age, and dummy‐coded race (whereby “other” was used as reference category) and education (0 = “Less than 12 years,” 1 = “In high school,” 2 = “High school graduate,” 3 = “Some college,” 4 = “College graduate,” 5 = “In graduate school”) as captured in the GPIPP dataset. At ZIP code‐level (Level 2), we controlled for median household income, gender ratio, median age, educational attainment, and racial composition. The ZIP code‐level data were retrieved from https://simplemaps.com/ and were based on 2020 U.S Census data.

### 2.3 Data analyses

To test our directional hypotheses, we implemented a three‐stage approach.

#### 2.3.1 Zero‐order correlations

First, we examined zero‐order correlations between personality traits and individual‐ and ZIP code‐level predictors. Comparing the effect sizes of the correlations indicated which land‐cover categories were associated with personality traits.

#### 2.3.2 Multilevel analyses

Second, we ran a series of multilevel regressions to account for the structure of our data by nesting individuals (Level 1) into ZIP codes (Level 2). To allow the association between the predictors and the outcome to differ across ZIP codes, we first ran a random intercept, random slope multilevel model, but the model failed to converge. Next, we ran a series of random intercept, fixed slope multilevel models.^3^

Level 1 predictor variables were derived from the GPIPP dataset and included gender, age, and race. Level 2 contained the ZIP code‐level predictors, namely the proportions of land‐cover across the nine categories, three climate indicators, (i.e., historical mean temperature, precipitation, and snowfall), and sociodemographic control variables (i.e., median household income, gender ratio, median age, educational attainment, and racial composition). For these models, we used the *lme4* package (Bates et al., 2007) and estimated with maximum likelihood to allow for model comparison. We *z*‐standardized all variables to ensure that coefficients are comparable across the models.

We built separate multilevel models for each Big Five outcome in a stepwise fashion. For each model, we first tested the broad environmental predictors: the nine land‐cover categories and the three climate indicators. Second, we added Level 1 control variables: gender, age, education, and dummy‐coded race. Third, we added the Level 2 control variables: median household income, gender ratio, median age, educational attainment, and racial composition. Lastly, we conducted further multilevel analyses as robustness tests to include the social‐class Level 1 variable in line with our preregistered strategy (Supplementary Information, Tables S3–S7).^4^ Unless otherwise stated, all associations remained stable across robustness tests.

#### 2.3.3 Random forest analyses

Finally, complementing our multilevel analyses, we employed supervised machine learning to accomplish three additional analytical objectives: first, using a random forest approach allowed the inclusion of all land‐cover categories, rather than eliminating one reference category (i.e., forest); second, it addressed multicollinearity and dependence of variance issues encountered with classical statistical approaches; third, it provided an estimate for the relative importance of the predictors in explaining each personality domain. We trained random forest models (Breiman, 2001) using the *ranger* package in R (Wright & Ziegler, 2015) for each Big Five domain on a 90% random sample of our data, reserving the remaining 10% test set for out‐of‐sample evaluation. A 10% random sample of the training set was used for hyperparameter tuning, a process that helped optimize configuration variables governing the training process itself. We mirrored the last stage of our main multilevel analyses to include the following predictors in these models: the ten land‐cover categories, the three climate indicators, the Level 1 and Level 2 control variables.

**FIGURE 1:**
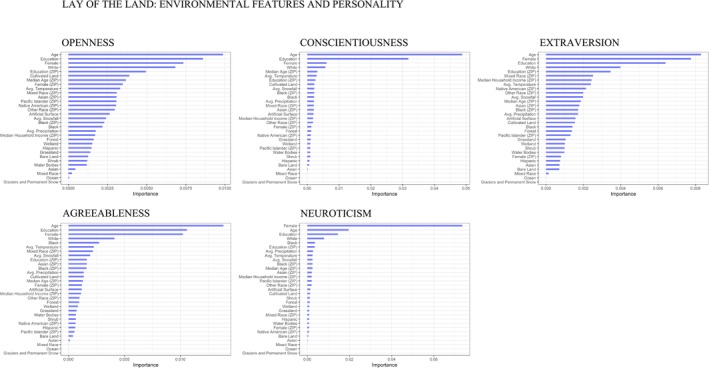
Random forest variance importance plots for (a) openness, (b) conscientiousness, (c) extraversion, (d) agreeableness, and (e) neuroticism.

For each random forest model, calculating variable importance allowed us to rank the relative importance of independent variables in predicting individual personality (Figure 1). Where variables have permutation importance scores below zero, predictions on the shuffled data were more accurate than the real data. Scores for each variable are model‐specific and do not reflect variables' intrinsic predictive value, making any cutoff point arbitrary. Nonetheless, to ease interpretation of the relatively large number of variables, we can follow a median split approach and consider, within each model, the top 15 ranked variables (out of the 31 total variables) as offering relatively stronger evidence, the lower ranking variables as offering relatively weaker evidence, and the variables with a value of zero as offering no evidence for the assessed relationship. The complete cached outputs depicting the raw variable importance scores are available online to further aid the interpretation of the analyses (Step 3 Notebook, https://osf.io/8j3en/).

Traditional impurity‐based feature importance can lead to inflated estimated importance of numerical features (Altmann et al., 2010), so we set *ranger* to calculate permutation importance as our variable importance metric (see Supplementary Information). Comparing model performance metrics for predicting each personality domain underscored differences in the out‐of‐sample predictions of personality traits from individual and group level. Our random forest model for openness showed the best performance, with a root mean square error (RMSE) score of .64, followed by models for agreeableness, conscientiousness, and neuroticism (RMSEs = .66, .67, and .79, respectively). Extraversion proved to be the most difficult trait to model (RMSE = .84).^5^

## 3 RESULTS

Overall, artificial surface and cultivated land ranked first and second among land‐cover variables when predicting personality traits. Glaciers and permanent snow was the only land‐cover category that showed negative relative importance across all the random forest models.

### 3.1 Openness to experience

We hypothesized that openness to experience would be positively associated with artificial surface (H1a), water bodies (H1b), wetland (H1c), and oceans (H1d). Openness to experience was positively associated with artificial surface, as indicated by the zero‐order correlations (*r* = .083; Table 1). This association remained consistent (i.e., maintained its significance and directionality across all steps of analysis) across the multilevel models that controlled for several Level 1 and 2 variables (Table 2) and across further robustness tests (Supplementary Information, Table S3). Artificial surface ranked 15th relative to the rest of predictors, providing relatively stronger evidence for the relationship between openness and urban spaces (Figure 1a).

**TABLE 1:** Zero‐order Pearson's correlations between land‐cover categories, zip code‐level predictors, and personality traits.

	Openness	Conscientiousness	Extraversion	Agreeableness	Neuroticism
Grassland	**−.016** [−.017, −.015]	**−.007** [−.008, −.005]	**−.004** [−.006, −.003]	**−.007** [−.008, −.005]	**−.001** [−.002, .001] ***(.377)***
Cultivated land	**−.077** [−.079, −.0762]	**.009** [.008, .010]	**.007** [.006, .008]	**.013** [.012, .013]	**.012** [.011, .013]
Forest	**−.036** [.037, .035]	**.010** [.008, .011]	**−.001** [−.002, .001] ***(.271)***	**.012** [.011, .013]	**.013** [.012, .014]
Shrub	**.003** [.002, .004]	**−.002** [−.003, −.001]	**−.008** [−.010, −.007]	**−.007** [−.009, −.006]	**−.007** [−.008, −.006]
Wetland	**−.021** [.022, .020]	**.016** [.015, .017]	**.007** [.006, .008]	**.019** [.017, .020]	**−.005** [−.006, −.004]
Water bodies	**.011** [.009, .012]	**−.001** [−.002, .001] ***(.245)***	**.003** [.002, .005]	**−.002** [−.003, −.001] ***(.008)***	**.000** [−.001, .002] ***(.596)***
Artificial Surface	**.083** [.082, .085]	**−.013** [−.014, −.012]	**−.003** [−.004, −.001]	**−.017** [−.018, −.015]	**−.013** [−.014, −.012]
Bare Land	**−.000** [−.002, .001] ***(.652)***	**−.001** [−.003, −.001] ***(.016)***	**−.004** [−.005, −.002]	**−.004** [−.005, −.003]	**.000** [−.001, .001] ***(.865)***
Glaciers and Permanent Snow	**.000** [−.001, .002] ***(.544)***	**−.000** [−.002, .001] ***(.528)***	**−.002** [−.002, −.001] ***(.005)***	**−.000** [−.001, .001] ***(.638)***	**.001** [−.001, .001] ***(.187)***
Ocean	**.011** [.010, .013]	**−.001** [−.002, −.000] ***(.066)***	**.001** [−.000, .002] ***(.102)***	**−.005** [−.006, −.003]	**−.001** [−.002, −.001] ***(.335)***
Education	**.124** [.122, .125]	**.195** [.193, .196]	**−.031** [−.033, −.029]	**.057** [.055, .058]	**−.057** [−.059, −.056]
Female	**−.084** [−.085, −.082]	**.077** [.075, .078]	**.057** [.055, .058]	**.118** [.117, .120]	**.204** [.203, .206]
Age	**.082** [.081, .084]	**.212** [.211, .213]	**−.039** [−.040, −.037]	**.094** [.092, .095]	**−.091** [−.092, −.089]
Asian	**.004** [.003, .006]	**−.030** [−.032, −.030]	**−.023** [−.025, −.022]	**−.023** [−.025, −.022]	**.006** [.005, .008]
Hispanic	**−.007** [−.008, .005]	**−.019** [−.021, −.018]	**.017** [.016, .019]	**.003** [.001, .005]	**−.014** [−.015, −.012]
Black	**.000** [−.002, .001] ***(.912)***	**.070** [.069, .072]	**.017** [.016, .019]	**.089** [.087, .090]	**−.084** [−.085, −.082]
White	**−.013** [−.015, −.012]	**−.016** [−.017, −014]	**−.016** [−.018, −.014]	**−.051** [−.055, −.049]	**.067** [.066, .068]
Mixed	**−.008** [−.010, −.006]	**−.019** [−.021, −.017]	**−.012** [−.014, −.010]	**.000** [−.001, .002] ***(.789)***	**.000** [−.001, .002] ***(.810)***
Education (ZIP)	**.079** [.078, .080]	**−.018** [−.020, −.017]	**.017** [.016, .018]	**−.028** [−.030, −.027]	**−.019** [−.020, −.018]
Female (ZIP)	**.010** [.008, .011]	**.007** [.006, .009]	**.005** [.004, .006]	**.016** [.015, .017]	**−.002** [−.004, −.001]
Median Age (ZIP)	**−.015** [−.016, −.014]	**−.014** [−.015, −.013]	**.001**[−.000, .002] ***(.132)***	**−.013** [−.014, −.011]	**.020** [.019, .021]
Median Income (ZIP)	**.030** [.029, .031]	**−.029** [−.030, −.028]	**.014** [.013, .016]	**−.028** [−.030, −.027]	**−.009** [−.010, −.008]
Asian (ZIP)	**.045** [.044, .046]	**−.032** [−.033, −.031]	**−.011** [−.012, −.009]	**−.025** [−.027, −.024]	**−.003** [−.004, −.002]
White (ZIP)	**−.048** [−.050, −.047]	**−.018** [−.019, −.017]	**.015** [.014, .016]	**−.020** [−.022, −.019]	**.029** [.028, .030]
Black (ZIP)	**.023** [.022, .024]	**.045** [.044, .046]	**−.003** [−.004, −.002]	**.040** [.039, .042]	**−.032** [−.034, −.031]
Native American (ZIP)	**−.007** [−.008, −.006]	**−.005** [−.006, −.004]	**−.008** [−.009, −.007]	**−.002** [−.003, −.001] ***(.009)***	**.002** [.001, .003] ***(.001)***
Pacific Islander (ZIP)	**.007** [.006, .009]	**−.003** [−.004, −.002]	**−.011** [−.012, −.010]	**−.004** [−.005, −.003]	**−.006** [−.008, −.005]
Other (ZIP)	**.028** [.026, .029]	**−.017** [−.018, −.016]	**−.016** [−.017, −.015]	**−.004** [−.005, −.003]	**−.003** [−.004, −.002]
Multiple races (ZIP)	**.040** [.038, .041]	**−.007** [−.008, −.005]	**−.019** [−.020, −.018]	**−.018** [−.020, −.017]	**−.004** [−.006, −.003]
Snowfall	**−.020** [−.022, −.019]	**−.015** [−.016, −.014]	**.002** [.001, .003] ***(.034)***	**−.012** [−.015, −.013]	**−.015** [−.014, −.016]
Temperature	**.035** [.034, .036]	**.015** [.013, .016]	**.001** [.000, .002] ***(.034)***	**.012** [.010, .013]	**−.020** [−.022, −.019]
Precipitation	**−.012** [−.014, −.011]	**.017** [.016, .018]	**.007** [.006, .007]	**.014** [.013, .015]	**.010** [.009, .011]

*Note*: *N* = 2,605,181. All correlations were significant at *p* < .001 unless otherwise stated in round parentheses. Values in square brackets indicate 95% confidence intervals.

**TABLE 2:** Results from multilevel models for openness to experience, *N* = 2,690,878, *N*_zip_ = 29,017.

DV = Openness	Model 0		Model 1		Model 2		Model 3		Model 4		Model 5	
	Estimates	95% CI	Estimates	95% CI	Estimates	95% CI	Estimates	95% CI	Estimates	95% CI	Estimates	95% CI
Intercept	−.0363***	−.0393, −.0334	−.0624***	−.0652, −.0597	−.0467***	−.0497, −.0438	−.0648***	−.0675, −.0620	−.0616***	−.0643, −.0590	−.0730***	−.0757, −.0703
Grassland			−.0033	−.0068, .0003			−.0058**	−.0096, −.0021	−.0068***	−.0104, −.0032	−.0072***	−.0106, −.0039
Cultivated Land			−.0490***	−.0530, −.0450			−.0500***	−.0542, −.0457	−.0444***	−.0485, −.0402	−.0280***	−.0319, −.0241
Wetland			−.0064***	−.0092, −.0035			−.0116***	−.0146, −.0086	−.0105***	−.0134, −.0076	−.0084***	−.0111, −.0058
Water Bodies			.0109***	.0081, .0136			.0108***	.0080, .0135	.0100***	.0074, .0126	.0050***	.0026, .0073
Artificial Surface			.0566***	.0534, .0598			.0496***	.0460, .0532	.0469***	.0435, .0504	.0236***	.0201, .0270
Shrub			.0153***	.0120, .0185			.0066***	.0027, .0106	.0050**	.0012, .0088	.0067***	.0033, .0102
Bare Land			.0049**	.0018, .0080			.0046**	.0015, .0077	.0035*	.0005, .0064	.0035*	.0008, .0062
Glaciers and Permanent Snow			.0017	−.0017, .0051			.0024	−.0010, .0058	.0025	−.0008, .0058	.0027	−.0005, .0059
Ocean			.0131***	.0103, .0158			.0117***	.0090, .0145	.0103***	.0076, .0129	.0054***	.0030, .0078
Snowfall					.0286***	.0238, .0334	.0077***	.0034, .0121	.0093***	.0051, .0135	.0078***	.0039, .0116
Temperature					.0640***	.0598, .0682	.0215***	.0173, .0257	.0223***	.0184, .0263	.0234***	.0198, .0270
Precipitation					−.0211***	−.0239, −.0182	−.0084***	−.0116, −.0052	−.0089***	−.0120, −.0058	−.0084***	−.0114, −.0054
*Individual Level Controls*												
Education									.0383***	.0368, .0399	.0365***	.0350, .0380
Gender									−.0742***	−.0754, −.0730	−.0738***	−.0750, −.0726
White									−.0803***	−.0829, −.0777	−.0820***	−.0846, −.0794
Asian									−.0322***	−.0337, −.0307	−.0332***	−.0347, −.0317
Hispanic									−.0583***	−.0602, −.0563	−.0580***	−.0600, −.0560
Mixed									−.0266***	−.0279, −.0252	−.0268***	−.0281, −.0254
Black									−.0530***	−.0551, −.0509	−.0539***	−.0560, −.0517
Age									.0416***	.0401, .0431	.0424***	.0409, .0439
*ZIP Level Controls*												
Median Age (ZIP)											.0268***	.0233, .0303
Female (ZIP)											−.0109***	−.0159, −.0060
Education (ZIP)											.0889***	.0852, .0926
Median Household Income (ZIP)											−.0319***	−.0353, −.0286
Black (ZIP)											.0160***	.0135, .0185
Asian (ZIP)											−.0045***	−.0066, −.0023
Native American (ZIP)											−.0168***	−.0216, −.0121
Pacific Islander (ZIP)											.0020	−.0044, .0083
Other (ZIP)											.0241***	.0216, .0266
Mixed (ZIP)											.0190***	.0147, .0234
*Model Fit*												
Marginal *R* ^2^/Conditional *R* ^2^	.000/.027		.009/.026		.003/.028		.010/.026		.021/.035		.025/.035	
Adjusted ICC	.027		.017		.025		.017		.014		.010	
AIC	7,062,532.102		7,057,314.476		7,061,426.596		7,057,214.151		7,028,213.495		7,025,717.965	

*Note*: The adjusted ICC captures Level‐2 random effects.

* *p* < .05

** *p* < .01

*** *p* < .001.

We expected openness to be positively associated with water‐related categories (H1b‐H1d), yet this relationship proved more complex. Openness positively correlated with oceans (*r* = .011) and water bodies (*r* = .011), and these associations remained robust across all multilevel analyses (Table 2). Water bodies and oceans ranked low (27th, 30th) suggesting that these categories explain relatively little variability in openness.^6^

In contrast to oceans and water bodies, wetlands capture marshes and swamps, which are an amalgam of both water bodies and vegetation. Contrary to our hypothesis, openness was negatively correlated to the proportion of wetland (*r* = −.021), and this association maintained its directionality and significance across all multilevel analyses, yet wetland ranked low (22nd) in its relative importance.

We ventured beyond our hypotheses and explored additional bivariate relationships. In so doing, we uncovered a negative association between cultivated land and openness (*r* = −.077), which remained robust across all multilevel models. Cultivated land ranked 6th in its relative importance in predicting openness, supporting the exploratory results emerging from the multilevel models. Next, we found a negative association between openness to experience and grassland (*r* = −.016), a category that captures prairies, primarily (Reese et al., 2016), yet random forest models positioned grassland low in its importance (24th). We did not identify consistent associations between openness to experience and the remaining land‐cover categories (shrub, bare land, glaciers, and permanent snow).

We further sought to replicate the relationship between temperature and openness to experience. Our analyses revealed a positive correlation between average temperature and openness (*r* = .035), which remained robust across all multilevel models (Table 2). Average temperature ranked high in its relative importance when predicting openness (9th).

### 3.2 Conscientiousness

We did not formulate any hypotheses regarding the relationship between conscientiousness and land‐cover categories. Exploratory analyses yielded a robust negative association between conscientiousness and artificial surface, captured by zero‐order correlations and multilevel analyses (*r* = −.013; Table 3). When predicting conscientiousness, artificial surface ranked 15th (Figure 1b), providing further support for this association.

**TABLE 3:** Results from multilevel models for conscientiousness, *N* = 2,690,878, *N*_zip_ = 29,017.

DV = Conscientiousness	Model 0		Model 1		Model 2		Model 3		Model 4		Model 5	
	Estimates	95% CI	Estimates	95% CI	Estimates	95% CI	Estimates	95% CI	Estimates	95% CI	Estimates	95% CI
Intercept	.0022	−.0001, .0045	.0042***	.0017, .0068	−.0008	−.0032, .0015	.0014	−.0011, .0039	.0156***	.0133, .0178	.0173***	.0148, .0197
Grassland			−.0141***	−.0173, −.0109			−.0070***	−.0104, −.0036	−.0026	−.0056, .0004	−.0002	−.0031, .0028
Cultivated Land			−.0063***	−.0100, −.0026			.0027	−.0012, .0066	.0222***	.0187, .0257	.0142***	.0107, .0177
Wetland			.0131***	.0105, .0157			.0082***	.0054, .0109	.0111***	.0087, .0136	.0095***	.0071, .0118
Water Bodies			−.0050***	−.0075, −.0025			−.0057***	−.0082, −.0033	−.0057***	−.0078, −.0035	−.0034**	−.0055, −.0014
Artificial Surface			−.0105***	−.0135, −.0076			−.0063***	−.0095, −.0030	−.0172***	−.0201, −.0144	−.0100***	−.0130, −.0070
Shrub			−.0088***	−.0117, −.0058			−.0022	−.0058, .0013	−.0033*	−.0064, −.0001	−.0049**	−.0079, −.0018
Bare Land			−.0015	−.0043, .0013			−.0001	−.0029, .0027	−.0009	−.0034, .0015	−.0009	−.0033, .0015
Glaciers and Permanent Snow			−.0007	−.0040, .0026			−.0012	−.0045, .0021	−.0005	−.0036, .0026	.0003	−.0027, .0034
Ocean			.0000	−.0025, .0025			−.0019	−.0044, .0005	−.0037***	−.0059, −.0015	−.0015	−.0036, .0006
Snowfall					−.0046*	−.0085, −.0008	−.0048*	−.0088, −.0009	.0046**	.0011, .0081	.0016	−.0018, .0050
Temperature					.0089***	.0056, .0122	.0104***	.0067, .0141	.0023	−.0010, .0055	.0008	−.0024, .0039
Precipitation					.0194***	.0172, .0216	.0152***	.0123, .0181	.0031*	.0006, .0056	−.0064***	−.0090, −.0038
*Individual Level Controls*												
Education									.1691***	.1677, .1706	.1699***	.1684, .1714
Gender									.0605***	.0593, .0617	.0603***	.0591, .0615
White									.0104***	.0079, .0129	.0095***	.0070, .0120
Asian									−.0156***	−.0170, −.0141	−.0147***	−.0162, −.0133
Hispanic									.0152***	.0133, .0171	.0148***	.0128, .0167
Mixed									−.0048***	−.0061, −.0035	−.0047***	−.0060, −.0034
Black									.0639***	.0619, .0660	.0607***	.0587, .0628
Age									.1560***	.1545, .1574	.1557***	.1542, .1571
*ZIP Level Controls*												
Median Age (ZIP)											−.0156***	−.0187, −.0125
Female (ZIP)											−.0011	−.0055, .0034
Education (ZIP)											−.0285***	−.0317, −.0254
Median Household Income (ZIP)											.0149***	.0119, .0178
Black (ZIP)											.0085***	.0063, .0107
Asian (ZIP)											−.0089***	−.0107, −.0070
Native American (ZIP)											−.0175***	−.0219, −.0131
Pacific Islander (ZIP)											.0105***	.0050, .0160
Other (ZIP)											−.0161***	−.0182, −.0139
Mixed (ZIP)											−.0159***	−.0198, −.0120
*Model Fit*												
Marginal *R* ^2^/Conditional *R* ^2^	.000/.012		.001/.012		.001/.012		.001/.012		.091/.098		.092/.098	
Adjusted ICC	.012		.011		.011		.011		.008		.007	
AIC	7,091,888.869		7,091,692.183		7,091,446.548		7,091,426.239		6,866,640.446		6,865,836.939	

*Note*: The adjusted ICC captures Level‐2 random effects.

* *p* < .05

** *p* < .01

*** *p* < .001.

Conscientiousness was also positively associated with wetland across all multilevel models (Table 3) and zero‐order correlations (*r* = .016). However, wetland ranked 22nd, explaining less variability in conscientiousness compared with the rest of the predictors.

When accounting for individual and ZIP code‐level sociodemographic correlates, conscientiousness was positively associated with cultivated land, yet this relationship was not consistent across all models (Table 3, Model 1 and 3). Cultivated land ranked high (8th) in predicting conscientiousness.^7^ All remaining associations between conscientiousness and land‐cover were inconsistent across analytical approaches.

### 3.3 Extraversion

We hypothesized that extraversion would be positively associated with artificial surface (H2a), water bodies (H2b), wetland (H2c), and oceans (H2d). Across the multilevel models, extraversion was positively associated with artificial surface (Table 4), yet this relationship did not maintain its significance across all robustness tests (Supplementary Information, Table S5, Model 4). Furthermore, zero‐order correlations indicated a negative, albeit small, link between extraversion and artificial surface (*r =* −.003, *p* < .001), providing mixed support for our hypothesis. Artificial surface ranked 16th in their importance when predicting extraversion (Figure 1c), offering only modest evidence for the hypothesis that extraversion is associated with urban spaces.

**TABLE 4:** Results from multilevel models for extraversion, *N* = 2,690,878, *N*_zip_ = 29,017.

DV = Extraversion	Model 0		Model 1		Model 2		Model 3		Model 4		Model 5	
	Estimates	95% CI	Estimates	95% CI	Estimates	95% CI	Estimates	95% CI	Estimates	95% CI	Estimates	95% CI
Intercept	−.0102***	−.0121, −.0082	−.0112***	−.0134, −.0090	−.0097***	−.0118, −.0077	−.0110***	−.0132, −.0088	−.0136***	−.0159, −.0114	−.0202***	−.0227, −.0178
Grassland			−.0010	−.0038, .0018			.0005	−.0024, .0035	.0014	−.0016, .0044	−.0005	−.0034, .0024
Cultivated Land			.0114***	.0082, .0147			.0144***	.0109, .0178	.0122***	.0088, .0157	.0112***	.0078, .0146
Wetland			.0057***	.0034, .0079			.0033**	.0009, .0057	.0022	−.0002, .0046	.0042***	.0019, .0065
Water Bodies			.0056***	.0035, .0077			.0056***	.0034, .0077	.0064***	.0043, .0085	.0037***	.0017, .0057
Artificial Surface			.0052***	.0027, .0077			.0058***	.0030, .0086	.0070***	.0042, .0098	.0049***	.0020, .0078
Shrub			−.0076***	−.0101, −.0050			−.0062***	−.0093, −.0032	−.0079***	−.0110, −.0049	−.0087***	−.0117, −.0058
Bare Land			−.0029*	−.0053, −.0004			−.0026*	−.0050, −.0001	−.0025*	−.0049, −.0000	−.0010	−.0033, .0013
Glaciers and Permanent Snow			−.0028	−.0060, .0003			−.0030	−.0061, .0002	−.0027	−.0059, .0004	−.0022	−.0053, .0009
Ocean			.0032**	.0011, .0054			.0028*	.0006, .0049	.0039***	.0018, .0061	.0023*	.0002, .0043
Snowfall					.0115***	.0082, .0148	.0125***	.0091, .0159	.0107***	.0073, .0141	.0062***	.0029, .0095
Temperature					.0078***	.0050, .0106	.0106***	.0074, .0137	.0083***	.0052, .0115	.0117***	.0087, .0147
Precipitation					.0064***	.0045, .0083	.0033**	.0009, .0057	−.0012	−.0036, .0012	−.0056***	−.0080, −.0031
*Individual Level Controls*												
Education									−.0067***	−.0082, −.0051	−.0079***	−.0095, −.0064
Gender									.0628***	.0616, .0641	.0631***	.0619, .0644
White									−.0044***	−.0070, −.0018	−.0075***	−.0101, −.0048
Asian									−.0330***	−.0345, −.0314	−.0337***	−.0353, −.0322
Hispanic									.0030**	.0010, .0050	.0036***	.0016, .0056
Mixed									−.0181***	−.0195, −.0167	−.0181***	−.0195, −.0167
Black									.0113***	.0092, .0134	.0130***	.0109, .0152
Age									−.0115***	−.0130, −.0099	−.0110***	−.0125, −.009
*ZIP Level Controls*												
Median Age (ZIP)											−.0066***	−.0096, −.0036
Female (ZIP)											.0035	−.0009, .0079
Education (ZIP)											.0200***	.0170, .0231
Median Household Income (ZIP)											.0131***	.0103, .0158
Black (ZIP)											−.0078***	−.0099, −.0056
Asian (ZIP)											−.0068***	−.0086, −.0051
Native American (ZIP)											−.0117***	−.0161, −.0072
Pacific Islander (ZIP)											−.0012	−.0065, .0041
Other (ZIP)											−.0087***	−.0108, −.0066
Mixed (ZIP)											−.0207***	−.0245, −.0170
*Model Fit*												
Marginal *R* ^2^/Conditional *R* ^2^	.000/.006		.000/.006		.000/.006		.000/.006		.006/.012		.007/.012	
Adjusted ICC	.006		.006		.006		.006		.006		.005	
AIC	7,099,461.977		7,099,369.627		7,099,413.201		7,099,346.313		7,085,330.161		7,084,088.522	

*Note*: The adjusted ICC captures Level‐2 random effects.

* *p* < .05

** *p* < .01

*** *p* < .001.

Extraversion was positively associated with water bodies (*r* = .003) and oceans across multilevel models, yet the bivariate correlation with oceans did not reach significance. Extraversion was also positively correlated with wetland (*r* = .007), yet this positive association was not consistent across all multilevel models (Table 4, Model 4). Wetland ranked 22nd among the predictors of extraversion, offering relatively weak evidence for an association between the two variables.^8^ Water bodies and oceans ranked 24th and 30th, respectively, in their importance as derived from random forest models.

Lastly, exploratory correlations and multilevel analyses revealed a positive association between extraversion and cultivated land (*r* = .007) and a negative association between extraversion and shrub (*r* = −.008). Cultivated land ranked 17th, whilst shrub ranked 18th in its relative importance, providing only modest evidence for these associations.

The associations between extraversion and the remaining land‐cover categories (grassland, bare land, glaciers, and permanent snow) were not consistent across models.

### 3.4 Agreeableness

We did not have any prior hypotheses about the relationship between agreeableness and land‐cover categories. Exploratory analyses revealed a positive association between wetland and agreeableness (*r* = .019), which remained robust across all analyses. Furthermore, agreeableness was negatively associated with artificial surfaces (*r* = −.017), shrub (*r* = −.007), and ocean (*r* = −.005); these relationships remained substantially the same across multilevel analyses (Table 5) and zero‐order correlations (Table 1), and subsequent robustness checks (Supplementary Information, Table S6). Variable‐importance plots provided only modest support for the relative importance of artificial surface, shrub, and ocean in predicting agreeableness, ranking 16th, 23rd, and 30th, respectively, in their relative performance.^9^

**TABLE 5:** Results from multilevel models for agreeableness, *N* = 2,690,878, *N*_zip_ = 29,017.

DV = Agreeableness	Model 0	Model 1		Model 2			Model 3			Model 4		Model 5	
	Estimates	95% CI	Estimates	95% CI	Estimates		95% CI	Estimates	95% CI	Estimates	95% CI	Estimates	95% CI
Intercept	−.0019	−.0040, .0003	.0016	−.0007, .0040	−.0050***	−.0073, −.0028	−.0019	−.0043, .0005	.0093***	.0070, .0116		.0098***	.0073, .0124
Grassland			−.0166***	−.0196, −.0136			−.0129***	−.0161, −.0097	−.0101***	−.0131, −.0070		−.0078***	−.0109, −.0048
Cultivated Land			−.0076***	−.0111, −.0041			−.0029	−.0066, .0008	.0064***	.0029, .0100		.0001	−.0035, .0037
Wetland			.0142***	.0118, .0167			.0098***	.0072, .0124	.0092***	.0068, .0117		.0083***	.0059, .0108
Water Bodies			−.0057***	−.0080, −.0034			−.0064***	−.0087, −.0041	−.0042***	−.0064, −.0021		−.0022*	−.0044, −.0001
Artificial Surface			−.0147***	−.0174, −.0119			−.0145***	−.0175, −.0114	−.0242***	−.0271, −.0212		−.0198***	−.0229, −.0167
Shrub			−.0152***	−.0180, −.0124			−.0146***	−.0180, −.0113	−.0155***	−.0187, −.0123		−.0164***	−.0195, −.0133
Bare Land			−.0042**	−.0068, −.0015			−.0034*	−.0060, −.0007	−.0039**	−.0064, −.0014		−.0039**	−.0064, −.0015
Glaciers and Permanent Snow			−.0006	−.0038, .0026			−.0006	−.0038, .0026	−.0001	−.0032, .0031		.0005	−.0026, .0036
Ocean			−.0054***	−.0077, −.0031			−.0072***	−.0096, −.0049	−.0060***	−.0083, −.0038		−.0040***	−.0062, −.0018
Snowfall					−.0113***	−.0149, −.0076	−.0117***	−.0154, −.0080	−.0064***	−.0099, −.0028		−.0088***	−.0123, −.0053
Temperature					.0043**	.0012, .0074	.0086***	.0051, .0120	−.0025	−.0058, .0008		−.0047**	−.0080, −.0015
Precipitation					.0177***	.0156, .0198	.0064***	.0037, .0090	−.0023	−.0048, .0003		−.0084***	−.0111, −.0058
*Individual Level Controls*													
Education									.0681***	.0666, .0697		.0688***	.0673, .0703
Gender									.0987***	.0975, .1000		.0985***	.0973, .0997
White									.0088***	.0062, .0114		.0090***	.0064, .0115
Asian									−.0083***	−.0098, −.0068		−.0077***	−.0092, −.0061
Hispanic									.0313***	.0294, .0333		.0311***	.0291, .0331
Mixed									.0099***	.0085, .0112		.0101***	.0087, .0115
Black									.0788***	.0767, .0809		.0770***	.0748, .0791
Age									.0723***	.0708, .0738		.0721***	.0706, .0736
*ZIP Level Controls*													
Median Age (ZIP)												−.0133***	−.0165, −.0101
Female (ZIP)												.0216***	.0170, .0262
Education (ZIP)												−.0206***	−.0239, −.0174
Median Household Income (ZIP)												.0117***	.0087, .0147
Black (ZIP)												.0040***	.0017, .0062
Asian (ZIP)												−.0035***	−.0054, −.0016
Native American (ZIP)												.0016	−.0029, .0061
Pacific Islander (ZIP)												.0063*	.0006, .0120
Other (ZIP)												−.0072***	−.0094, −.0049
Mixed (ZIP)												−.0273***	−.0313, −.0233
*Model Fit*													
Marginal *R* ^2^/Conditional *R* ^2^	.000/.010		.001/.009		.001/.010		.001/.010		.032/.039			.033/.039	
Adjusted ICC	.010		.009		.009		.009		.007			.007	
AIC	7,095,233.817		7,094,761.961		7,094,769.476		7,094,498.560		7,019,025.333			7,018,618.771	

*Note*: The adjusted ICC captures Level‐2 random effects.

* *p* < .05

** *p* < .01

*** *p* < .001.

The relationships between agreeableness and the remaining land‐cover categories (cultivated land, water bodies, bare land, glaciers, and permanent snow) were inconsistent across analytical approaches.

### 3.5 Neuroticism

We did not formulate any hypotheses regarding the relationship between neuroticism and land‐cover categories. Exploratory analyses revealed neuroticism to be negatively associated with wetland (*r* = −.005) and artificial surface (*r* = −.013), associations that remained robust across both correlational and multilevel analyses (Table 6). Artificial surface ranked 16th and wetland ranked 22nd when predicting neuroticism, providing only modest support for these associations (Figure 1e).^10^

**TABLE 6:** Results from multilevel models for neuroticism, *N* = 2,690,878, *N*_zip_ = 29,017.

DV = Neuroticism	Model 0		Model 1		Model 2		Model 3		Model 4		Model 5	
	Estimates	95% CI	Estimates	95% CI	Estimates	95% CI	Estimates	95% CI	Estimates	95% CI	Estimates	95% CI
Intercept	.0167***	.0145, .0188	.0285***		.0240***	.0218, .0261	.0310***	.0287, .0334	.0125***	.0102, .0147	.0207***	.0183, .0231
Grassland			−.0107***	−.0137, −.0077			−.0083***	−.0115, −.0052	−.0083***	−.0113, −.0054	−.0051***	−.0080, −.0021
Cultivated Land			−.0030	−.0064, .0004			−.0016	−.0052, .0021	−.0101***	−.0135, −.0067	−.0109***	−.0143, −.0075
Wetland			−.0122***	−.0147, −.0098			−.0077***	−.0102, −.0051	−.0079***	−.0103, −.0055	−.0086***	−.0109, −.0063
Water Bodies			−.0034**	−.0057, −.0011			−.0032**	−.0055, −.0010	−.0045***	−.0066, −.0024	−.0026*	−.0046, −.0006
Artificial Surface			−.0271***	−.0297, −.0244			−.0204***	−.0234, −.0174	−.0037*	−.0065, −.0009	.0035*	.0006, .0064
Shrub			−.0147***	−.0175, −.0120			−.0063***	−.0096, −.0030	−.0046**	−.0077, −.0015	−.0027	−.0056, .0003
Bare Land			−.0010	−.0036, .0016			−.0007	−.0033, .0019	.0013	−.0011, .0037	.0009	−.0014, .0033
Glaciers and Permanent Snow			.0007	−.0025, .0039			.0001	−.0031, .0033	−.0005	−.0036, .0026	−.0012	−.0042, .0019
Ocean			−.0036**	−.0059, −.0013			−.0023*	−.0046, −.0000	−.0020	−.0042, .0001	−.0008	−.0029, .0012
Snow Fall					−.0054**	−.0089, −.0019	−.0006	−.0043, .0030	−.0030	−.0064, .0004	−.0012	−.0045, .0021
Temperature					−.0273***	−.0303, −.0242	−.0163***	−.0197, −.0130	−.0049**	−.0080, −.0017	−.0077***	−.0108, −.0047
Precipitation					.0133***	.0112, .0153	.0078***	.0052, .0104	.0122***	.0098, .0147	.0176***	.0151, .0201
*Individual Level Controls*												
Education									−.0445***	−.0460, −.0430	−.0430***	−.0445, −.0415
Gender									.2245***	.2233, .2257	.2242***	.2230, .2254
White									.0310***	.0285, .0335	.0322***	.0296, .0347
Asian									.0144***	.0129, .0158	.0145***	.0130, .0159
Hispanic									−.0104***	−.0124, −.0085	−.0114***	−.0133, −.0094
Mixed									.0030***	.0017, .0043	.0027***	.0014, .0040
Black									−.0671***	−.0692, −.0651	−.0666***	−.0687, −.0645
Age									−.0774***	−.0788, −.0759	−.0780***	−.0794, −.0765
*ZIP Level Controls*												
Median Age (ZIP)											.0091***	.0061, .0122
Female (ZIP)											.0023	−.0021, .0067
Education (ZIP)											−.0227***	−.0257, −.0196
Median Household Income (ZIP)											−.0158***	−.0186, −.0129
Black (ZIP)											−.0084***	−.0106, −.0063
Asian (ZIP)											.0102***	.0085, .0120
Native American (ZIP)											−.0016	−.0060, .0028
Pacific Islander (ZIP)											−.0165***	−.0218, −.0111
Other (ZIP)											.0032**	.0011, .0053
Mixed (ZIP)											.0145***	.0106, .0183
*Model Fit*												
Marginal *R* ^2^/Conditional *R* ^2^	.000/.009		.001/.009		.001/.009		.001/.009		.067/.074		.068/.073	
Adjusted ICC	.009		.008		.008		.008		.007		.006	
AIC	7,098,030.560		7,097,381.832		7,097,472.276		7,097,236.536		6,927,768.904		6,926,783.211	

*Note*: The adjusted ICC captures Level‐2 random effects.

* *p* < .05

** *p* < .01

*** *p* < .001.

## 4 DISCUSSION

In the current study, we inventoried landscapes, operationalized using satellite imagery of ten types of land‐cover in the United States, and found several robust associations between landscapes and personality traits.

### 4.1 Openness to experience

We found a positive association between openness and the proportion of artificial surface, a category that captures built‐up areas composed of buildings or roads. This relationship remained largely unchanged when tested against several individual and ZIP code‐level demographic variables. When using a machine learning validation approach, artificial surface was in the top half of predictors for openness, when benchmarked against a conservative set of sociodemographic controls and alternative ecological predictors.

Openness was positively associated with oceans, yet oceans ranked second to last in their variable importance when predicting openness in random forest models. Our land‐cover taxonomy solely captures in‐land variation, so the oceans category failed to comprehensively incorporate all ZIP codes that are simply bordering oceans. Therefore, we conducted additional analyses using a different operationalization of oceans that records whether or not a ZIP code is positioned on the coast (NOAA, 2022; https://coast.noaa.gov/digitalcoast/data/enow.html). This follow‐up analysis indeed found elevated openness in coastal regions.

Water bodies, which capture lakes, rivers, or bays, were found to be positively associated with openness to experience across multilevel analysis, yet water bodies ranked low among predictors of openness (27th), providing, at best, mixed evidence for this association.

Wetland was negatively associated with openness to experience, with zero‐order correlations and multilevel analyses yielding robust associations, yet the random forests variable importance rankings placed wetland on the 22nd position among the predictors of openness, providing only modest support for this association. Somewhat surprisingly, this finding is distinct from that between openness and oceans or water bodies, considering that U.S. wetlands are predominantly located on the coast or are lake‐adjacent. This result may capture the link between wetlands and commercial practices. In the United States, despite being less prevalent than other landscapes, wetlands are one of the most productive ecosystems as many industries harvest fish that depend on the wetland's ecosystem (Rewa, 2007). Arguably, wetlands capture distinct geographies and attract distinct personality traits compared to regions around lakes, rivers, or oceans.

Our exploratory analyses revealed that residents of areas containing cultivated land had lower levels of openness, with cultivated land ranking 6th among all predictors of openness. The negative association with openness can perhaps be explained by the nature of agricultural practices. Agriculture requires time to be spent locally, close to the cultivated land and, in turn, can diminish the time to explore and travel.

### 4.2 Conscientiousness

Cultivated land was positively associated with conscientiousness when accounting for individual and ZIP code‐level sociodemographic correlates. This association was supported by random forest models which placed cultivated land among the first eight predictors of conscientiousness. Land cultivation requires industriousness, diligence, and following strict timelines, all traits associated with conscientiousness.

Our additional multilevel analyses that included the coastal variable found that coastal regions consistently have lower levels of conscientiousness compared to non‐coastal regions. Due to their exploratory nature, these findings should be further consolidated, potentially using different datasets and regions outside the United States.

### 4.3 Extraversion

A positive association between extraversion and artificial surface emerged across multilevel analyses, when accounting for the nested structure of the data, and was moderately supported by its relative importance ranking (16th) in the random forest analysis. This association was unstable across further robustness checks, failing to provide robust empirical support for our hypothesis (H2a). Future studies should consider using different datasets and methodological approaches to further probe this association.

Extraversion was positively associated with water bodies and wetlands but their relative importance when predicting extraversion (24th, 22nd) provided only modest support for this association (H2b, H2c). Similarly, extraversion was positively associated with oceans across the multilevel models. However, the zero‐order correlations, robustness checks and the association between openness and the coastal variable did not reveal consistent associations. Oceans also ranked second to last among the predictors for extraversion. Overall, results failed to provide evidence that extraversion is higher in coastal areas (H2d).

### 4.4 Agreeableness

Exploratory zero‐order correlations and multilevel analyses revealed that agreeableness is positively associated with wetland and negatively associated with artificial surface, shrub, and oceans. However, in the random forest models, these variables were all positioned in the second half of agreeableness predictors, suggesting their relative unimportance in explaining agreeableness. Our additional multilevel analyses revealed that coastal areas exhibit lower levels of agreeableness compared to non‐coastal areas.

### 4.5 Neuroticism

Neuroticism was negatively associated with wetlands and artificial surface across both zero‐order correlations and multilevel analyses. However, importance rankings positioned both variables in the second half of all predictors of neuroticism, thus providing only weak support for these associations. Additional multilevel analyses revealed higher levels of neuroticism in coastal regions compared to non‐coastal regions.

### 4.6 Implications

Geographical clustering of personality traits has been long documented, bringing to light a macroperspective to personality research (Allik & McCrae, 2004; Oishi & Graham, 2010; Rentfrow, 2010; Schmitt et al., 2007). Ecological theory suggests one factor that contributes to this variation in personality is the physical environment in which individuals reside. We aimed to further this line of inquiry and assess whether—and if so how—personality is associated with landscape. To accomplish this goal, we modeled a large U.S. self‐report sample alongside land‐cover satellite imagery, linking ecological variables to individual‐level outcomes. To the best of our knowledge, this represents the first empirical attempt to quantify landscape and systematically assess its relationship with personality traits.

We note that the observed effects were consistently very small. The highest zero‐order correlations between ZIP code‐level land‐cover and personality in the current study were the associations between openness and artificial surface (*r* = .083) and cultivated land (*r* = −.077), respectively. Importantly though, these associations received robust empirical support across our three‐pronged analytical procedure and the strength of the associations is comparable to—and in some cases twice as strong as—the correlations between openness and established sociodemographic correlates of openness (i.e., education [*r* = .079], median household income [*r* = .030], racial composition [*r* = −.045 to .040]).^11^ These correlations are also consistent with (a) the associations that one may theoretically expect between a distal, ecological influence (e.g., land‐cover) and a complex, multiply determined outcome (e.g., personality) and (b) the associations that have been empirically observed for other ecological variables—such as temperature clemency (Wei et al., 2017) and physical topography (Götz et al., 2020)—in relation to personality. Nevertheless, a traditional view on effect sizes would likely dismiss such small correlations as irrelevant. As part of a re‐emerging debate, scholars have recently argued small effects in psychological science (a) may actually be a norm, rather than an exception (Gignac & Szodorai, 2016; Götz et al., 2022) and (b) can have important downstream consequences when considered at scale and over time (Funder & Ozer, 2019; Matz et al., 2017). The latter appears plausible in the present context given that (a) distal ecological influences, such as landscapes may affect large groups of individuals who share the same environmental milieu (i.e., accumulation at scale; Lu et al., 2018; Oishi, 2014) and (b) personality continuously influences important personal decisions and life outcomes (i.e., accumulation over time; Ozer & Benet‐Martinez, 2006; Soto, 2019). However, caution is warranted. Simply because all small effects could theoretically matter in practice, not all will (Anvari et al., 2022). While, based on theoretical grounds, accumulation appears more likely to occur in the present context than counteracting processes (e.g., habituation, homeostasis; Anvari et al., 2022), we are unable to address this question empirically with the current dataset and call on future research to investigate whether and how ecological factors shaping individual personality translate into consequential real‐world outcomes.

### 4.7 Limitations

While this design had methodological advantages, it also had limitations. First, the present study had a cross‐sectional, correlational design, which did not assess causality. Despite the effort to control for several potential confounding variables, our study may have captured spurious associations due to, for example, measurement error. Through this study's design, we cannot disambiguate which of the three drivers—selective migration, social influence, and environmental influence (Rentfrow et al., 2008)—may be at play. Nonetheless, we largely find that landscapes more conducive to human activity tend to be associated with distinct personality distributions, potentially indicative of how both social and environmental influence shape regional variation in personality traits. Some of the associations between landscape and personality—such as the findings for artificial surface or cultivated land—could also be the result of a reverse causality process, whereby people use land according to their personality so that personality changes the landscape (Florida, 2014).

Second, our findings do not speak to the direct person‐level associations between personality and landscape. Future studies should consider a person‐level design that collects landscape perceptions, time of residence, and personality data from participants.

Third, we tested the association between personality and landscape in one Western country that may not generalize to other countries. Past research has demonstrated how associations between personality and ecological factors such as climate or topography exhibit both similarities and differences when tested across different samples (e.g., Wei et al., 2017; Xu et al., 2022). For example, prior studies uncovered positive associations between terrain elevation and openness across the United States (Götz et al., 2020) and China (Xu et al., 2022). Yet differences also emerged: agreeableness and elevation were positively associated in the US (Götz et al., 2020) but negatively associated in China (Xu et al., 2022). In a similar vein, we expect our findings to generalize to other samples, but we also acknowledge that idiosyncratic socioecological mechanisms may translate to distinct findings (e.g., Talhelm et al., 2014). Future studies should seek to replicate our current findings in other contexts to evaluate the extent to which these results are limited to the U.S. context.

## 5 CONCLUSION

Taken together, our results suggest that landscape categories that incur more human interventions such as cultivated land and artificial surface are significantly associated with human personality. The relationships between personality traits and landscapes that provide the backdrop for human activity to a lesser extent, such as grassland, wetland, water bodies and oceans, are less stable across analytic approaches. Lastly, personality traits do not appear to be significantly associated with landscapes that are freer from human interference, such as forests, bare lands, shrub, or glaciers and permanent snow.

While there is much that remains to be addressed by more targeted, individual‐level investigations, the present study may offer insights into why regions differ in their personality traits. As natural environments change around the world, landscape, and climatic modifications could further impact variations in personality traits.

## AUTHOR CONTRIBUTIONS

IEM: conceptualization, analyses, writing, reviewing, editing, project administration. GSG: conceptualization, analyses, writing, reviewing and editing. TE: conceptualization, data collection, reviewing and editing. WK: data collection. SDG: data collection, reviewing and editing. Jeff Potter: data collection. PJR: conceptualization, writing, reviewing and editing. FMG: conceptualization, data collection, writing, reviewing and editing.

## FUNDING INFORMATION

IEM is supported by the Economic and Social Research Council PhD scholarship and the Nokia Bell Labs PhD scholarship no. ES/J500033/. GSG is supported by the Bill & Melinda Gates Foundation through a Gates Cambridge Scholarship [OPP1144]. FMG is supported by a Hampton Fund Research Grant. The funders had no role in designing the study, data collection, or data analysis.

## CONFLICT OF INTEREST STATEMENT

The author(s) declared no potential conflict of interest with respect to the research.

## ETHICS STATEMENT

The personality data obtained from the Gosling‐Potter Internet Personality Project received ethical approval from the institutional ethics boards at the University of California and the University of Texas.

## Supporting information


Appendix S1

## Endnotes

^1^
All analyses were performed at ZIP Code Tabulation Areas (ZCTA) level as defined by the U.S. Census Bureau (2011). We refer to these areal representations as “ZIP codes” throughout the current article.

^2^
We conducted a preliminary analysis to investigate whether any of the land‐cover categories are highly correlated (i.e., *r* > .70) and could be better summarized as joint categories. This pre‐analysis consistently detected correlations below .45 between the ten land‐covercategories (Supplementary Information, Table S1), suggesting that we could proceed using the land‐cover categories as individual variables.

^3^
The required sample size for multilevel analysis was reached as our data included more than 200 ZIP codes (i.e., Level 2 units; Arend & Schäfer, 2019; Maas & Hox, 2005).

^4^
Despite various transformations attempts, the multilevel models violated several assumptions of linear regressions, including the normality and constant variance assumptions (see Supplementary Information for further details). While some of these assumptions can be relaxed when dealing with a large sample size, violations can impact the robustness of estimates (Luo et al., 2021; Maas & Hox, 2004; Schielzeth et al., 2020). The models should be interpreted alongside the random forest analyses which do not rest upon such assumptions.

^5^
Lower RMSE scores indicate better model fit.

^6^
Our operationalization of oceans does not comprehensively capture areas that border oceans, but rather oceans that are surrounded by land. To address this limitation, we ran additional exploratory multilevel analyses where we replaced the oceans variable with the coast‐adjacent variable and found that—in line with H1d—openness was higher in coastal regions than in non‐coastal regions (Supplementary Information, Table S3).

^7^
Additional exploratory multilevel analyses revealed that coastal ZIP codes have lower levels of conscientiousness than non‐coastal ZIP codes (Supplementary Information, Table S4).

^8^
We went beyond our preregistered analyses and tested whether residents of coastal ZIP codes have higher levels of extraversion than residents of non‐coastal ZIP codes. Multilevel analyses failed to identify a consistent and significant association between extraversion and coastal areas (Supplementary Information, Table S5).

^9^
Additional exploratory multilevel analyses suggested that coastal ZIP codes have lower levels of agreeableness than non‐coastal ZIP codes (Supplementary Information, Table S4).

^10^
Additional exploratory multilevel models found coastal ZIP codes to have higher levels of neuroticism compared to non‐coastal ZIP codes.

^11^
Using the methodology outlined by Diedenhofen and Musch (2015), we assessed if the correlations between openness and land‐cover categories are indeed significantly larger in magnitude than those between openness and sociodemographic correlates. Pairwise comparisons revealed that the correlation between (1) openness and artificial surface is significantly larger in magnitude than the correlations between (2) openness and education, (3) openness and median household income, and (4) openness and racial composition.
