# Supplementary material for: The lay of the land: Associations between environmental features and personality
Source: J Pers. 2023 Apr 8;92(1):88–110. doi: 10.1111/jopy.12822 (PMC10952236; doi:10.1111/jopy.12822)
Supplement: Supplementary file 1 — Appendix S1 [file JOPY-92-88-s001.docx]

**­­The lay of the land: Associations between environmental features and personality**

**Supplementary Information**

Ioana Elisabeta Militaru^1^, Gregory Serapio-García^1^, Tobias Ebert^2^, Wenyuan Kong^3^, Samuel D. Gosling^4^, Jeff Potter^5^, Peter J. Rentfrow^6^, Friedrich M. Götz^7^

^1^University of Cambridge, UK­­

^2^University of Mannheim, Germany

^3^Peking University, China

^4^University of Texas at Austin, USA

^5^University of Melbourne, Australia

^6^Atof Inc., USA

^7^University of British Columbia, Canada

**Correspondence**:

Ioana Elisabeta Militaru, Department of Psychology, University of Cambridge, Cambridge, CB23RQ, UK. Email: [iem24@cam.ac.uk](mailto:iem24@cam.ac.uk) ORCID: 0000-0003-4856-1598

Friedrich M. Götz, Department of Psychology, Unversity of British Columbia, 2136 West Mall, Vancouver, BC V6T 1Z4, Canada. Email: [friedrich.goetz@ubc.ca](mailto:friedrich.goetz@ubc.ca) ORCID: 0000-0001-8900-6844

**2. Method**

**2.2. Measures**

| **Table S1.** *Correlations between the land-usage categories.* | | | | | | | | | | | |
| --- | --- | --- | --- | --- | --- | --- | --- | --- | --- | --- | --- |
|  | *1* | | *2* | *3* | *4* | *5* | *6* | *7* | *8* | *9* | *10* |
| *1. Grassland* | |  |  |  |  |  |  |  |  |  |  |
| *2. Cultivated land* | -0.116 | |  |  |  |  |  |  |  |  |  |
| *3. Forest* | -0.217 | | -0.368 |  |  |  |  |  |  |  |  |
| *4. Wetland* | -.090 | | -.070 | -.064 |  |  |  |  |  |  |  |
| *5. Water bodies* | -.026 | | -0.134 | -.049 | 0.170 |  |  |  |  |  |  |
| *6. Artificial surface* | -0.153 | | -0.443 | -0.389 | -0.124 | .022 |  |  |  |  |  |
| *7. Shrub* | 0.122 | | -0.213 | -0.166 | -.085 | -.071 | -0.136 |  |  |  |  |
| *8. Bare land* | .099 | | -.089 | -.048 | -.021 | .029 | -.036 | 0.136 |  |  |  |
| *9. Glaciers and permanent snow* | .001 *(.875)* | | -.016 *(.003)* | .020 | -.007 *(.221)* | -.004 *(.459)* | -.012 *(.036)* | .009 *(.113)* | .070 |  |  |
| *10 Ocean* | -.015 *(.008)* | | -.086 | -.031 | .043 | .008 *(.128)* | .025 | -.020 | .040 | -.002 *(.705)* |  |
| *Note*. All correlations were significant with *p<*.001 unless stated otherwise. Computed correlation used Pearson-method with listwise-deletion. | | | | | | | | | | | |

| **Table S2*.*** *Mean ZIP code level proportions of land-usage categories across U.S. ZIP codes and their description.* | | |
| --- | --- | --- |
| **Category** | **Proportion** | **Description** |
| Grassland | .084 | Lands covered by natural grass with cover of over 10%. |
| Cultivated land | 0.294 | Lands used for agriculture, horticulture, and gardens. |
| Forest | 0.306 | Lands covered with trees, with vegetation cover of over 30%. |
| Wetland | .038 | Lands covered with wetland plants and water bodies. |
| Water bodies | .019 | Water bodies in the land area. |
| Artificial surface | 0.194 | Lands modified by human activities. |
| Shrub | .057 | Lands covered with shrubs with cover over 30%. |
| Bare land | .004 | Lands with vegetation cover lower than 10%. |
| Glaciers and permanent snow | <.001 | Lands covered by permanent snow, glaciers and icecap |
| Ocean | .003 | Water bodies outside the land area. |
| Tundra | 0 | Lands covered by lichen, moss, hardy perennial herb and shrubs in the polar regions. |
| *Note.* Descriptions of the categories were retrieved from the National Catalogue Service for Geographic Information ( [www.webmap.cn](http://www.webmap.cn)). | | |

**2.3. Data Analyses**

**2.3.1. Multilevel analyses**

All analyses were performed in R using the package *lme4* (Bates et al., 2007) and diagnostics were ran using the *HLMdiag* package (Loy & Hofmann, 2014). Three main assumptions underlie (linear) multilevel models: linear relationships, homoscedasticity, and a normal distribution of residuals both at the individual level and group level (Maas & Hox, 2004). We used three visual checks to establish adherence to these assumptions for the five final models (Model 5 of each trait): residuals versus fitted plots, histograms of residuals and QQ plots. All plots along with the associated code are uploaded on the project OSF page (<https://osf.io/8j3en/?view_only=1366a87131b84d46bebcc184665d9b06>).

We ran additional multilevel analyses to ensure the robustness of our results. First, in line with our preregistered analysis plan, we added the social class variable. Second, we replaced the ocean variable originating from the land use dataset with a binary variable indexing the coastal and non-coastal U.S. ZIP codes collected from the National Ocean Economics Program (NOEP; [www.oceaneconomics.org](http://www.oceaneconomics.org)). The use of the coastal variable addressed the limitation of the land-usage dataset which only captures inland variation. As such, the oceans category only captures pixels that are within the boundaries of a ZIP code and fails to capture all ZIP codes that border oceans.

**2.3.2. Random forest analyses**

**Model specification*.*** For model tuning and resampling, we used the *ranger* package’s computational engine for random forest regression (Wright & Ziegler, 2017), with number of trees set to 1,000 and split rule set to *maxstat*. For model testing on holdout data, we modified this engine specification to include calculation permutation importance. Since the calculation of permutation importance is a computationally intensive process, creating a separate engine specification for testing allowed us to reduce compute time significantly for tuning and resampling where calculating permutation importance was not necessary.

**Hyperparameter tuning and model selection.** To optimize predictive performance for each Big Five trait, we tuned the hyperparameters *mtry* and *min_n*—representing the number of predictors randomly sampled at each split and the minimum number of data points, respectively—for each model using a 10-fold split of a 10% sample of the training data. To do so while maximizing efficiency, we conducted a Latin hypercube grid search via racing with ANOVA models using the *dials* (Kuhn & Frick, 2022) and *finetune* (Kuhn, 2022) packages in R. The grid search process yielded optimal model parameter combinations for each Big Five trait that minimized cross-validated RMSE.

| **Table S3.** Results from robustness Multilevel Modelling tests for Openness to experience, *N*=1,529,503, *N_zip_*=28,013. | | | | | | | | | | | | | |
| --- | --- | --- | --- | --- | --- | --- | --- | --- | --- | --- | --- | --- | --- |
|  | **Model 0** | | **Model 1** | | **Model 2** | | **Model 3** | | **Model 4** | | **Model 5** | |  |
| *DV****=Openness*** | *Estimates* | *95% CI* | *Estimates* | *95% CI* | *Estimates* | *95% CI* | *Estimates* | *95% CI* | *Estimates* | *95% CI* | *Estimates* | *95% CI* |  |
| Intercept | -.0208 ^***^ | -.0241 – -.0175 | -.0575 ^***^ | -.0607 – -.0544 | -.0284 ^***^ | -.0317 – -.0251 | -.0587 ^***^ | -.0619 – -.0555 | -.0901 ^***^ | -.0931 – -.0870 | -0.1042 ^***^ | -0.1073 – -0.1010 |  |
| Grassland |  |  | -.0054 ^**^ | -.0095 – -.0014 |  |  | -.0087 ^***^ | -.0130 – -.0044 | -.0108 ^***^ | -.0148 – -.0067 | -.0078 ^***^ | -.0116 – -.0041 |  |
| Cultivated Land |  |  | -.0509 ^***^ | -.0555 – -.0464 |  |  | -.0536 ^***^ | -.0585 – -.0487 | -.0446 ^***^ | -.0492 – -.0400 | -.0264 ^***^ | -.0308 – -.0220 |  |
| Wetland |  |  | -.0146 ^***^ | -.0179 – -.0112 |  |  | -.0196 ^***^ | -.0232 – -.0160 | -.0159 ^***^ | -.0192 – -.0126 | -.0129 ^***^ | -.0159 – -.0098 |  |
| Water Bodies |  |  | .0067 ^***^ | .0037 – .0098 |  |  | .0068 ^***^ | .0037 – .0099 | .0058 ^***^ | .0029 – .0086 | .0021 | -.0005 – .0047 |  |
| Artificial Surface |  |  | .0606 ^***^ | .0571 – .0642 |  |  | .0532 ^***^ | .0492 – .0572 | .0468 ^***^ | .0431 – .0505 | .0227 ^***^ | .0190 – .0264 |  |
| Shrub |  |  | .0107 ^***^ | .0070 – .0145 |  |  | .0010 | -.0035 – .0056 | .0005 | -.0037 – .0047 | .0034 | -.0005 – .0073 |  |
| Bare Land |  |  | .0023 | -.0012 – .0058 |  |  | .0018 | -.0017 – .0054 | .0011 | -.0022 – .0044 | .0004 | -.0026 – .0034 |  |
| Glaciers and Permanent Snow |  |  | .0017 | -.0025 – .0059 |  |  | .0024 | -.0018 – .0066 | .0025 | -.0016 – .0065 | .0028 | -.0011 – .0067 |  |
| Coastal |  |  | .0206 ^***^ | .0175 – .0237 |  |  | .0215 ^***^ | .0184 – .0246 | .0188 ^***^ | .0159 – .0216 | .0149 ^***^ | .0123 – .0175 |  |
| Snow Fall |  |  |  |  | .0341 ^***^ | .0287 – .0395 | .0077 ^**^ | .0027 – .0126 | .0075 ^**^ | .0029 – .0121 | .0040 | -.0003 – .0082 |  |
| Average Temperature |  |  |  |  | .0602 ^***^ | .0553 – .0651 | .0192 ^***^ | .0144 – .0240 | .0184 ^***^ | .0140 – .0228 | .0170 ^***^ | .0129 – .0210 |  |
| Precipitation |  |  |  |  | -.0171 ^***^ | -.0203 – -.0138 | -.0100 ^***^ | -.0137 – -.0063 | -.0105 ^***^ | -.0139 – -.0071 | -.0098 ^***^ | -.0131 – -.0066 |  |
| **Individual Level Controls** | | | | | | | | | | | | | |
| Education |  |  |  |  |  |  |  |  | .0915 ^***^ | .0896 – .0935 | .0879 ^***^ | .0859 – .0898 |  |
| Social Class |  |  |  |  |  |  |  |  | .0218 ^***^ | .0202 – .0234 | .0199 ^***^ | .0182 – .0215 |  |
| Gender |  |  |  |  |  |  |  |  | -.0890 ^***^ | -.0906 – -.0875 | -.0883 ^***^ | -.0899 – -.0868 |  |
| White |  |  |  |  |  |  |  |  | -.0847 ^***^ | -.0880 – -.0814 | -.0862 ^***^ | -.0895 – -.0828 |  |
| Asian |  |  |  |  |  |  |  |  | -.0363 ^***^ | -.0384 – -.0342 | -.0374 ^***^ | -.0395 – -.0352 |  |
| Hispanic |  |  |  |  |  |  |  |  | -.0555 ^***^ | -.0580 – -.0530 | -.0553 ^***^ | -.0579 – -.0528 |  |
| Mixed |  |  |  |  |  |  |  |  | -.0264 ^***^ | -.0283 – -.0246 | -.0267 ^***^ | -.0285 – -.0248 |  |
| Black |  |  |  |  |  |  |  |  | -.0533 ^***^ | -.0559 – -.0507 | -.0549 ^***^ | -.0575 – -.0523 |  |
| Age |  |  |  |  |  |  |  |  | .0372 ^***^ | .0355 – .0390 | .0383 ^***^ | .0366 – .0401 |  |
| **ZIP Level Controls** | | | | | | | | | | | | | |
| Median Age (ZIP) |  |  |  |  |  |  |  |  |  |  | .0244 ^***^ | .0204 – .0284 |  |
| Female (ZIP) |  |  |  |  |  |  |  |  |  |  | -.0143 ^***^ | -.0199 – -.0088 |  |
| Education (ZIP) |  |  |  |  |  |  |  |  |  |  | 0.1026 ^***^ | .0986 – 0.1066 |  |
| Median Household Income (ZIP) |  |  |  |  |  |  |  |  |  |  | -.0510 ^***^ | -.0546 – -.0473 |  |
| Black (ZIP) |  |  |  |  |  |  |  |  |  |  | .0177 ^***^ | .0149 – .0205 |  |
| Asian (ZIP) |  |  |  |  |  |  |  |  |  |  | -.0014 | -.0037 – .0009 |  |
| Native (ZIP) |  |  |  |  |  |  |  |  |  |  | -.0170 ^***^ | -.0226 – -.0114 |  |
| Pacific (ZIP) |  |  |  |  |  |  |  |  |  |  | -.0030 | -.0101 – .0041 |  |
| Other (ZIP) |  |  |  |  |  |  |  |  |  |  | .0285 ^***^ | .0258 – .0313 |  |
| Mixed (ZIP) |  |  |  |  |  |  |  |  |  |  | .0134 ^***^ | .0086 – .0183 |  |
| **Model Fit** | | | | | | | | | | | | | |
| Adjusted ICC | .032 | | .020 | | .031 | | .020 | | .015 | | .010 | |  |
| Marginal R2 / Conditional R2 | .000 / .032 | | .011 / .031 | | .002 / .033 | | .012 / .031 | | .033 / .048 | | .039 / .049 | |  |
| AIC | 4311113.087 | | 4306195.177 | | 4310458.562 | | 4306141.672 | | 4274379.830 | | 4271970.113 | |  |
| ** p<.05   ** p<.01   *** p<.001* | | | | | | | | | | | | | |

| **Table S4.** Results from robustness Multilevel Modelling tests for Conscientiousness, *N*=1,529,503, *N_zip_*=28,013. | | | | | | | | | | | | |
| --- | --- | --- | --- | --- | --- | --- | --- | --- | --- | --- | --- | --- |
|  | **Model 0** | | **Model 1** | | **Model 2** | | **Model 3** | | **Model 4** | | **Model 5** | |
| *DV=****Conscientiousness*** | *Estimates* | *95% CI* | *Estimates* | *95% CI* | *Estimates* | *95% CI* | *Estimates* | *95% CI* | *Estimates* | *95% CI* | *Estimates* | *95% CI* |
| Intercept | 0.1539 ^***^ | 0.1515 – 0.1563 | 0.1562 ^***^ | 0.1535 – 0.1589 | 0.1480 ^***^ | 0.1455 – 0.1504 | 0.1528 ^***^ | 0.1501 – 0.1555 | .0760 ^***^ | .0734 – .0786 | .0836 ^***^ | .0807 – .0864 |
| Grassland |  |  | -.0101 ^***^ | -.0136 – -.0066 |  |  | -.0029 | -.0065 – .0008 | -.0019 | -.0052 – .0015 | .0007 | -.0026 – .0039 |
| Cultivated Land |  |  | -.0109 ^***^ | -.0149 – -.0069 |  |  | -.0023 | -.0065 – .0019 | .0168 ^***^ | .0129 – .0207 | .0062 ^**^ | .0023 – .0100 |
| Wetland |  |  | .0185 ^***^ | .0157 – .0214 |  |  | .0097 ^***^ | .0067 – .0127 | .0139 ^***^ | .0111 – .0167 | .0104 ^***^ | .0077 – .0131 |
| Water Bodies |  |  | -.0052 ^***^ | -.0078 – -.0026 |  |  | -.0058 ^***^ | -.0084 – -.0033 | -.0057 ^***^ | -.0081 – -.0034 | -.0027 ^*^ | -.0049 – -.0004 |
| Artificial Surface |  |  | -.0107 ^***^ | -.0137 – -.0077 |  |  | -.0092 ^***^ | -.0126 – -.0059 | -.0215 ^***^ | -.0246 – -.0184 | -.0118 ^***^ | -.0150 – -.0086 |
| Shrub |  |  | -.0058 ^***^ | -.0090 – -.0026 |  |  | -.0020 | -.0059 – .0018 | -.0021 | -.0056 – .0014 | -.0037 ^*^ | -.0071 – -.0004 |
| Bare Land |  |  | -.0011 | -.0041 – .0020 |  |  | .0005 | -.0024 – .0035 | .0009 | -.0018 – .0037 | .0007 | -.0019 – .0033 |
| Glaciers and Permanent Snow |  |  | -.0008 | -.0047 – .0031 |  |  | -.0012 | -.0051 – .0027 | -.0004 | -.0041 – .0033 | .0001 | -.0036 – .0037 |
| Coastal |  |  | -.0049 ^***^ | -.0075 – -.0024 |  |  | -.0057 ^***^ | -.0083 – -.0032 | -.0063 ^***^ | -.0087 – -.0040 | -.0028 ^*^ | -.0050 – -.0006 |
| Snow Fall |  |  |  |  | -.0033 | -.0074 – .0007 | -.0033 | -.0074 – .0009 | .0024 | -.0014 – .0062 | .0020 | -.0016 – .0057 |
| Average Temperature |  |  |  |  | .0195 ^***^ | .0160 – .0231 | .0201 ^***^ | .0162 – .0241 | .0110 ^***^ | .0073 – .0146 | .0078 ^***^ | .0044 – .0113 |
| Precipitation |  |  |  |  | .0182 ^***^ | .0159 – .0205 | .0152 ^***^ | .0122 – .0182 | .0017 | -.0010 – .0045 | -.0056 ^***^ | -.0083 – -.0028 |
| **Individual Level Controls** | | | | | | | | | | | | |
| Education |  |  |  |  |  |  |  |  | 0.1375 ^***^ | 0.1356 – 0.1394 | 0.1406 ^***^ | 0.1388 – 0.1425 |
| Social Class |  |  |  |  |  |  |  |  | .0702 ^***^ | .0687 – .0718 | .0724 ^***^ | .0708 – .0739 |
| Gender |  |  |  |  |  |  |  |  | .0580 ^***^ | .0565 – .0595 | .0573 ^***^ | .0558 – .0588 |
| White |  |  |  |  |  |  |  |  | -.0083 ^***^ | -.0114 – -.0052 | -.0087 ^***^ | -.0119 – -.0055 |
| Asian |  |  |  |  |  |  |  |  | -.0177 ^***^ | -.0197 – -.0156 | -.0164 ^***^ | -.0184 – -.0143 |
| Hispanic |  |  |  |  |  |  |  |  | .0154 ^***^ | .0130 – .0178 | .0143 ^***^ | .0119 – .0167 |
| Mixed |  |  |  |  |  |  |  |  | -.0040 ^***^ | -.0058 – -.0023 | -.0039 ^***^ | -.0057 – -.0022 |
| Black |  |  |  |  |  |  |  |  | .0655 ^***^ | .0630 – .0679 | .0612 ^***^ | .0587 – .0637 |
| Age |  |  |  |  |  |  |  |  | 0.1303 ^***^ | 0.1286 – 0.1320 | 0.1295 ^***^ | 0.1278 – 0.1312 |
| **ZIP Level Controls** | | | | | | | | | | | | |
| Median Age (ZIP) |  |  |  |  |  |  |  |  |  |  | -.0132 ^***^ | -.0167 – -.0097 |
| Female (ZIP) |  |  |  |  |  |  |  |  |  |  | .0038 | -.0011 – .0087 |
| Education (ZIP) |  |  |  |  |  |  |  |  |  |  | -.0508 ^***^ | -.0542 – -.0474 |
| Median Household Income (ZIP) |  |  |  |  |  |  |  |  |  |  | .0188 ^***^ | .0157 – .0219 |
| Black (ZIP) |  |  |  |  |  |  |  |  |  |  | .0085 ^***^ | .0061 – .0109 |
| Asian (ZIP) |  |  |  |  |  |  |  |  |  |  | -.0063 ^***^ | -.0083 – -.0044 |
| Native (ZIP) |  |  |  |  |  |  |  |  |  |  | -.0206 ^***^ | -.0257 – -.0155 |
| Pacific (ZIP) |  |  |  |  |  |  |  |  |  |  | .0095 ^**^ | .0035 – .0155 |
| Other (ZIP) |  |  |  |  |  |  |  |  |  |  | -.0145 ^***^ | -.0168 – -.0122 |
| Mixed (ZIP) |  |  |  |  |  |  |  |  |  |  | -.0087 ^***^ | -.0129 – -.0044 |
| **Model Fit** | | | | | | | | | | | | |
| Adjusted ICC | .012 | | .011 | | .011 | | .010 | | .008 | | .006 | |
| Marginal R^2^ / Conditional R^2^ | .000 / .012 | | .000 / .011 | | .001 / .012 | | .001 / .011 | | .074 / .082 | | .075 / .081 | |
| AIC | 4255574.109 | | 4255364.132 | | 4254980.980 | | 4254931.030 | | 4143100.506 | | 4141743.789 | |
| *Note*. Forest was excluded from analyses to avoid data dependability. ** p<.05   ** p<.01   *** p<.001* | | | | | | | | | | | | |

| **Table S5.** Results from robustness Multilevel Modelling tests for Extraversion, *N*=1,529,503, *N_zip_*=28,013. | | | | | | | | | | | | |
| --- | --- | --- | --- | --- | --- | --- | --- | --- | --- | --- | --- | --- |
|  | **Model 0** | | **Model 1** | | **Model 2** | | **Model 3** | | **Model 4** | | **Model 5** | |
| *DV=****Extraversion*** | *Estimates* | *95% CI* | *Estimates* | *95% CI* | *Estimates* | *95% CI* | *Estimates* | *95% CI* | *Estimates* | *95% CI* | *Estimates* | *95% CI* |
| Intercept | .0312 ^***^ | .0292 – .0333 | .0290 ^***^ | .0265 – .0314 | .0279 ^***^ | .0257 – .0301 | .0276 ^***^ | .0251 – .0300 | .0553 ^***^ | .0529 – .0578 | .0540 ^***^ | .0512 – .0568 |
| Grassland |  |  | -.0001 | -.0032 – .0030 |  |  | .0004 | -.0029 – .0036 | -.0025 | -.0056 – .0006 | -.0016 | -.0047 – .0016 |
| Cultivated Land |  |  | .0088 ^***^ | .0052 – .0124 |  |  | .0110 ^***^ | .0071 – .0148 | .0074 ^***^ | .0037 – .0110 | .0063 ^**^ | .0025 – .0101 |
| Wetland |  |  | .0093 ^***^ | .0067 – .0119 |  |  | .0036 ^**^ | .0009 – .0063 | .0026 ^*^ | .0000 – .0052 | .0041 ^**^ | .0016 – .0067 |
| Water Bodies |  |  | .0032 ^**^ | .0010 – .0055 |  |  | .0032 ^**^ | .0009 – .0055 | .0034 ^**^ | .0012 – .0055 | .0025 ^*^ | .0004 – .0047 |
| Artificial Surface |  |  | .0070 ^***^ | .0043 – .0097 |  |  | .0037 ^*^ | .0007 – .0068 | -.0007 | -.0036 – .0022 | .0036 ^*^ | .0005 – .0067 |
| Shrub |  |  | -.0048 ^***^ | -.0077 – -.0020 |  |  | -.0087 ^***^ | -.0121 – -.0053 | -.0113 ^***^ | -.0145 – -.0080 | -.0111 ^***^ | -.0143 – -.0079 |
| Bare Land |  |  | -.0024 | -.0051 – .0003 |  |  | -.0023 | -.0050 – .0004 | -.0022 | -.0048 – .0003 | -.0013 | -.0039 – .0012 |
| Glaciers and Permanent Snow |  |  | -.0036 | -.0074 – .0003 |  |  | -.0033 | -.0071 – .0006 | -.0029 | -.0067 – .0008 | -.0025 | -.0063 – .0012 |
| Coastal |  |  | .0004 | -.0018 – .0026 |  |  | .0006 | -.0016 – .0028 | .0026 ^*^ | .0005 – .0046 | .0022 ^*^ | .0001 – .0043 |
| Snow Fall |  |  |  |  | .0098 ^***^ | .0063 – .0134 | .0110 ^***^ | .0073 – .0147 | .0085 ^***^ | .0050 – .0120 | .0042 ^*^ | .0007 – .0077 |
| Average Temperature |  |  |  |  | .0178 ^***^ | .0146 – .0209 | .0203 ^***^ | .0168 – .0238 | .0129 ^***^ | .0096 – .0161 | .0136 ^***^ | .0103 – .0169 |
| Precipitation |  |  |  |  | .0029 ^**^ | .0009 – .0049 | -.0006 | -.0032 – .0020 | -.0053 ^***^ | -.0078 – -.0029 | -.0082 ^***^ | -.0108 – -.0056 |
| **Individual Level Controls** | | | | | | | | | | | | |
| Education |  |  |  |  |  |  |  |  | -.0238 ^***^ | -.0258 – -.0219 | -.0240 ^***^ | -.0260 – -.0220 |
| Social Class |  |  |  |  |  |  |  |  | 0.1146 ^***^ | 0.1130 – 0.1163 | 0.1131 ^***^ | 0.1115 – 0.1148 |
| Gender |  |  |  |  |  |  |  |  | .0630 ^***^ | .0615 – .0646 | .0631 ^***^ | .0616 – .0647 |
| White |  |  |  |  |  |  |  |  | -.0112 ^***^ | -.0145 – -.0079 | -.0144 ^***^ | -.0177 – -.0111 |
| Asian |  |  |  |  |  |  |  |  | -.0335 ^***^ | -.0356 – -.0314 | -.0339 ^***^ | -.0360 – -.0317 |
| Hispanic |  |  |  |  |  |  |  |  | .0088 ^***^ | .0063 – .0113 | .0086 ^***^ | .0060 – .0111 |
| Mixed |  |  |  |  |  |  |  |  | -.0175 ^***^ | -.0193 – -.0157 | -.0175 ^***^ | -.0193 – -.0156 |
| Black |  |  |  |  |  |  |  |  | .0136 ^***^ | .0110 – .0161 | .0149 ^***^ | .0123 – .0176 |
| Age |  |  |  |  |  |  |  |  | -.0511 ^***^ | -.0529 – -.0494 | -.0513 ^***^ | -.0531 – -.0495 |
| **ZIP Level Controls** | | | | | | | | | | | | |
| Median Age (ZIP) |  |  |  |  |  |  |  |  |  |  | -.0004 | -.0038 – .0030 |
| Female (ZIP) |  |  |  |  |  |  |  |  |  |  | .0099 ^***^ | .0050 – .0147 |
| Education (ZIP) |  |  |  |  |  |  |  |  |  |  | .0025 | -.0007 – .0057 |
| Median Household Income (ZIP) |  |  |  |  |  |  |  |  |  |  | .0088 ^***^ | .0058 – .0118 |
| Black (ZIP) |  |  |  |  |  |  |  |  |  |  | -.0089 ^***^ | -.0112 – -.0066 |
| Asian (ZIP) |  |  |  |  |  |  |  |  |  |  | -.0059 ^***^ | -.0078 – -.0041 |
| Native (ZIP) |  |  |  |  |  |  |  |  |  |  | -.0151 ^***^ | -.0203 – -.0100 |
| Pacific (ZIP) |  |  |  |  |  |  |  |  |  |  | .0039 | -.0019 – .0096 |
| Other (ZIP) |  |  |  |  |  |  |  |  |  |  | -.0067 ^***^ | -.0089 – -.0045 |
| Mixed (ZIP) |  |  |  |  |  |  |  |  |  |  | -.0155 ^***^ | -.0196 – -.0114 |
| **Model Fit** | | | | | | | | | | | | |
| Adjusted ICC | .005 | | .005 | | .005 | | .005 | | .004 | | .004 | |
| Marginal R^2^ / Conditional R^2^ | .000 / .005 | | .000 / .005 | | .000 / .005 | | .000 / .005 | | .019 / .023 | | .020 / .023 | |
| AIC | 4308052.906 | | 4308038.849 | | 4307934.602 | | 4307929.784 | | 4279484.081 | | 4279199.902 | |
| *Note*. Forest was excluded from analyses to avoid data dependability. ** p<.05   ** p<.01   *** p<.001* | | | | | | | | | | | | |

| **Table S6.** Results from robustness Multilevel Modelling tests for Agreeableness, , *N*=1,529,503, *N_zip_*=28,013. | | | | | | | | | | | | |
| --- | --- | --- | --- | --- | --- | --- | --- | --- | --- | --- | --- | --- |
|  | **Model 0** | | **Model 1** | | **Model 2** | | **Model 3** | | **Model 4** | | **Model 5** | |
| *DV=****Agreeableness*** | *Estimates* | *95% CI* | *Estimates* | *95% CI* | *Estimates* | *95% CI* | *Estimates* | *95% CI* | *Estimates* | *95% CI* | *Estimates* | *95% CI* |
| ntercept | .0502 ^***^ | .0478 – .0526 | .0559 ^***^ | .0533 – .0586 | .0458 ^***^ | .0434 – .0483 | .0528 ^***^ | .0502 – .0555 | .0190 ^***^ | .0165 – .0216 | .0226 ^***^ | .0197 – .0255 |
| Grassland |  |  | -.0141 ^***^ | -.0176 – -.0107 |  |  | -.0100 ^***^ | -.0136 – -.0064 | -.0075 ^***^ | -.0108 – -.0042 | -.0055 ^**^ | -.0089 – -.0022 |
| Cultivated Land |  |  | -.0087 ^***^ | -.0126 – -.0048 |  |  | -.0049 ^*^ | -.0091 – -.0007 | .0033 | -.0006 – .0071 | -.0036 | -.0075 – .0003 |
| Wetland |  |  | .0211 ^***^ | .0183 – .0239 |  |  | .0145 ^***^ | .0115 – .0175 | .0124 ^***^ | .0096 – .0151 | .0106 ^***^ | .0079 – .0133 |
| Water Bodies |  |  | -.0056 ^***^ | -.0081 – -.0030 |  |  | -.0061 ^***^ | -.0086 – -.0036 | -.0035 ^**^ | -.0058 – -.0012 | -.0016 | -.0039 – .0006 |
| Artificial Surface |  |  | -.0170 ^***^ | -.0200 – -.0140 |  |  | -.0178 ^***^ | -.0212 – -.0145 | -.0257 ^***^ | -.0288 – -.0226 | -.0190 ^***^ | -.0222 – -.0157 |
| Shrub |  |  | -.0138 ^***^ | -.0169 – -.0107 |  |  | -.0140 ^***^ | -.0177 – -.0102 | -.0149 ^***^ | -.0183 – -.0114 | -.0160 ^***^ | -.0194 – -.0126 |
| Bare Land |  |  | -.0032 ^*^ | -.0062 – -.0003 |  |  | -.0022 | -.0052 – .0007 | -.0025 | -.0052 – .0002 | -.0024 | -.0051 – .0002 |
| Glaciers and Permanent Snow |  |  | -.0006 | -.0046 – .0033 |  |  | -.0007 | -.0046 – .0032 | -.0001 | -.0039 – .0037 | .0003 | -.0034 – .0041 |
| Coastal |  |  | -.0122 ^***^ | -.0147 – -.0097 |  |  | -.0124 ^***^ | -.0149 – -.0099 | -.0093 ^***^ | -.0116 – -.0071 | -.0066 ^***^ | -.0089 – -.0044 |
| Snow Fall |  |  |  |  | -.0133 ^***^ | -.0174 – -.0093 | -.0125 ^***^ | -.0166 – -.0085 | -.0069 ^***^ | -.0107 – -.0031 | -.0094 ^***^ | -.0131 – -.0057 |
| Average Temperature |  |  |  |  | .0073 ^***^ | .0037 – .0109 | .0109 ^***^ | .0071 – .0148 | .0014 | -.0022 – .0049 | -.0009 | -.0044 – .0026 |
| Precipitation |  |  |  |  | .0180 ^***^ | .0157 – .0203 | .0079 ^***^ | .0050 – .0108 | -.0027 ^*^ | -.0054 – -.0001 | -.0094 ^***^ | -.0121 – -.0066 |
| **Individual Level Controls** | | | | | | | | | | | | |
| Education |  |  |  |  |  |  |  |  | .0268 ^***^ | .0248 – .0287 | .0289 ^***^ | .0269 – .0308 |
| Social Class |  |  |  |  |  |  |  |  | -.0094 ^***^ | -.0111 – -.0078 | -.0087 ^***^ | -.0104 – -.0071 |
| Gender |  |  |  |  |  |  |  |  | 0.1067 ^***^ | 0.1052 – 0.1083 | 0.1062 ^***^ | 0.1046 – 0.1077 |
| White |  |  |  |  |  |  |  |  | .0012 | -.0020 – .0045 | .0012 | -.0021 – .0045 |
| Asian |  |  |  |  |  |  |  |  | -.0076 ^***^ | -.0097 – -.0055 | -.0066 ^***^ | -.0087 – -.0044 |
| Hispanic |  |  |  |  |  |  |  |  | .0281 ^***^ | .0256 – .0306 | .0276 ^***^ | .0251 – .0301 |
| Mixed |  |  |  |  |  |  |  |  | .0120 ^***^ | .0101 – .0138 | .0123 ^***^ | .0105 – .0141 |
| Black |  |  |  |  |  |  |  |  | .0820 ^***^ | .0795 – .0846 | .0797 ^***^ | .0771 – .0823 |
| Age |  |  |  |  |  |  |  |  | .0825 ^***^ | .0808 – .0843 | .0819 ^***^ | .0801 – .0836 |
| **ZIP Level Controls** | | | | | | | | | | | | |
| Median Age (ZIP) |  |  |  |  |  |  |  |  |  |  | -.0098 ^***^ | -.0134 – -.0063 |
| Female (ZIP) |  |  |  |  |  |  |  |  |  |  | .0249 ^***^ | .0199 – .0299 |
| Education (ZIP) |  |  |  |  |  |  |  |  |  |  | -.0290 ^***^ | -.0324 – -.0256 |
| Median Household Income (ZIP) |  |  |  |  |  |  |  |  |  |  | .0165 ^***^ | .0133 – .0196 |
| Black (ZIP) |  |  |  |  |  |  |  |  |  |  | .0032 ^*^ | .0007 – .0056 |
| Asian (ZIP) |  |  |  |  |  |  |  |  |  |  | -.0044 ^***^ | -.0063 – -.0024 |
| Native (ZIP) |  |  |  |  |  |  |  |  |  |  | .0007 | -.0046 – .0059 |
| Pacific (ZIP) |  |  |  |  |  |  |  |  |  |  | .0067 ^*^ | .0007 – .0128 |
| Other (ZIP) |  |  |  |  |  |  |  |  |  |  | -.0084 ^***^ | -.0107 – -.0060 |
| Mixed (ZIP) |  |  |  |  |  |  |  |  |  |  | -.0273 ^***^ | -.0316 – -.0230 |
| **Model Fit** | | | | | | | | | | | | |
| Adjusted ICC | .010 | | .009 | | .010 | | .009 | | .006 | | .005 | |
| Marginal R^2^ / Conditional R^2^ | .000 / .010 | | .001 / .010 | | .001 / .011 | | .001 / .010 | | .032 / .038 | | .033 / .038 | |
| AIC | 4303512.379 | | 4302933.537 | | 4303042.284 | | 4302631.412 | | 4257720.246 | | 4257213.961 | |
| *Note*. Forest was excluded from analyses to avoid data dependability*. * p<.05   ** p<.01   *** p<.001* | | | | | | | | | | | | |

| **Table S7.** Results from robustness Multilevel Modelling tests for Neuroticism, , *N*=1,529,503, *N_zip_*=28,013. | | | | | | | | | | | | |
| --- | --- | --- | --- | --- | --- | --- | --- | --- | --- | --- | --- | --- |
|  | **Model 0** | | **Model 1** | | **Model 2** | | **Model 3** | | **Model 4** | | **Model 5** | |
| *DV=****Neuroticism*** | *Estimates* | *95% CI* | *Estimates* | *95% CI* | *Estimates* | *95% CI* | *Estimates* | *95% CI* | *Estimates* | *95% CI* | *Estimates* | *95% CI* |
| Intercept | -.0425 ^***^ | -.0448 – -.0401 | -.0277 ^***^ | -.0304 – -.0251 | -.0347 ^***^ | -.0371 – -.0323 | -.0254 ^***^ | -.0280 – -.0227 | -.0101 ^***^ | -.0125 – -.0076 | -.0068 ^***^ | -.0096 – -.0040 |
| Grassland |  |  | -.0119 ^***^ | -.0153 – -.0085 |  |  | -.0088 ^***^ | -.0124 – -.0053 | -.0050 ^**^ | -.0082 – -.0018 | -.0047 ^**^ | -.0079 – -.0015 |
| Cultivated Land |  |  | .0035 | -.0004 – .0074 |  |  | .0068 ^**^ | .0026 – .0109 | -.0040 ^*^ | -.0077 – -.0003 | -.0025 | -.0063 – .0013 |
| Wetland |  |  | -.0171 ^***^ | -.0199 – -.0143 |  |  | -.0094 ^***^ | -.0123 – -.0065 | -.0112 ^***^ | -.0139 – -.0086 | -.0108 ^***^ | -.0135 – -.0082 |
| Water Bodies |  |  | -.0004 | -.0029 – .0021 |  |  | -.0003 | -.0028 – .0022 | -.0010 | -.0032 – .0012 | -.0009 | -.0031 – .0012 |
| Artificial Surface |  |  | -.0267 ^***^ | -.0296 – -.0237 |  |  | -.0169 ^***^ | -.0202 – -.0137 | .0042 ^**^ | .0013 – .0072 | .0049 ^**^ | .0018 – .0081 |
| Shrub |  |  | -.0151 ^***^ | -.0182 – -.0120 |  |  | -.0026 | -.0063 – .0011 | -.0011 | -.0044 – .0022 | .0007 | -.0026 – .0040 |
| Bare Land |  |  | -.0019 | -.0048 – .0011 |  |  | -.0014 | -.0043 – .0015 | -.0003 | -.0029 – .0023 | -.0001 | -.0027 – .0025 |
| Glaciers and Permanent Snow |  |  | .0003 | -.0036 – .0043 |  |  | -.0005 | -.0044 – .0034 | -.0012 | -.0049 – .0025 | -.0017 | -.0054 – .0020 |
| Coastal |  |  | .0045 ^***^ | .0020 – .0070 |  |  | .0034 ^**^ | .0009 – .0058 | .0033 ^**^ | .0012 – .0055 | .0023 ^*^ | .0001 – .0044 |
| Snow Fall |  |  |  |  | -.0054 ^**^ | -.0093 – -.0014 | .0006 | -.0034 – .0047 | -.0015 | -.0051 – .0021 | .0003 | -.0033 – .0039 |
| Average Temperature |  |  |  |  | -.0334 ^***^ | -.0369 – -.0300 | -.0221 ^***^ | -.0259 – -.0183 | -.0067 ^***^ | -.0101 – -.0033 | -.0068 ^***^ | -.0102 – -.0034 |
| Precipitation |  |  |  |  | .0140 ^***^ | .0117 – .0162 | .0102 ^***^ | .0073 – .0130 | .0171 ^***^ | .0146 – .0196 | .0220 ^***^ | .0193 – .0246 |
| **Individual Level Controls** | | | | | | | | | | | | |
| Education |  |  |  |  |  |  |  |  | -.0357 ^***^ | -.0377 – -.0338 | -.0352 ^***^ | -.0371 – -.0332 |
| Social Class |  |  |  |  |  |  |  |  | -.0998 ^***^ | -0.1013 – -.0982 | -.0991 ^***^ | -0.1007 – -.0975 |
| Gender |  |  |  |  |  |  |  |  | 0.2111 ^***^ | 0.2095 – 0.2126 | 0.2110 ^***^ | 0.2095 – 0.2125 |
| White |  |  |  |  |  |  |  |  | .0463 ^***^ | .0431 – .0495 | .0470 ^***^ | .0437 – .0502 |
| Asian |  |  |  |  |  |  |  |  | .0149 ^***^ | .0128 – .0169 | .0140 ^***^ | .0119 – .0161 |
| Hispanic |  |  |  |  |  |  |  |  | -.0117 ^***^ | -.0142 – -.0093 | -.0122 ^***^ | -.0147 – -.0097 |
| Mixed |  |  |  |  |  |  |  |  | .0019 ^*^ | .0000 – .0037 | .0013 | -.0005 – .0032 |
| Black |  |  |  |  |  |  |  |  | -.0705 ^***^ | -.0730 – -.0680 | -.0686 ^***^ | -.0712 – -.0661 |
| Age |  |  |  |  |  |  |  |  | -.0726 ^***^ | -.0743 – -.0709 | -.0729 ^***^ | -.0746 – -.0711 |
| **ZIP Level Controls** | | | | | | | | | | | | |
| Median Age (ZIP) |  |  |  |  |  |  |  |  |  |  | .0090 ^***^ | .0055 – .0124 |
| Female (ZIP) |  |  |  |  |  |  |  |  |  |  | .0010 | -.0039 – .0058 |
| Education (ZIP) |  |  |  |  |  |  |  |  |  |  | -.0036 ^*^ | -.0069 – -.0003 |
| Median Household Income (ZIP) |  |  |  |  |  |  |  |  |  |  | -.0089 ^***^ | -.0119 – -.0058 |
| Black (ZIP) |  |  |  |  |  |  |  |  |  |  | -.0074 ^***^ | -.0097 – -.0050 |
| Asian (ZIP) |  |  |  |  |  |  |  |  |  |  | .0099 ^***^ | .0080 – .0118 |
| Native (ZIP) |  |  |  |  |  |  |  |  |  |  | .0022 | -.0030 – .0073 |
| Pacific (ZIP) |  |  |  |  |  |  |  |  |  |  | -.0197 ^***^ | -.0256 – -.0138 |
| Other (ZIP) |  |  |  |  |  |  |  |  |  |  | .0043 ^***^ | .0021 – .0066 |
| Mixed (ZIP) |  |  |  |  |  |  |  |  |  |  | .0105 ^***^ | .0063 – .0146 |
| **Model Fit** | | | | | | | | | | | | |
| Adjusted ICC | .009 | | .008 | | .008 | | .008 | | .005 | | .005 | |
| Marginal R^2^ / Conditional R^2^ | .000 / .009 | | .001 / .009 | | .001 / .009 | | .001 / .009 | | .073 / .078 | | .074 / .078 | |
| AIC | 4332733.634 | | 4332100.704 | | 4332037.036 | | 4331829.725 | | 4219018.753 | | 4218878.877 | |
| *Note*. Forest was excluded from analyses to avoid data dependability. ** p<.05   ** p<.01   *** p<.001* | | | | | | | | | | | | |
|  | | | | | | | | | | | | |

**References**

Bates, D., Sarkar, D., Bates, M. D., & Matrix, L. (2007). The lme4 package. *R Package Version*, *2*(1), 74.

Kuhn, M. (2022). *finetune: Additional functions for model tuning* (0.2.0.9000). https://github.com/tidymodels/finetune

Kuhn, M., & Frick, H. (2022). *dials: Tools for creating tuning parameter values* (0.1.1). <https://github.com/tidymodels/dials>

Loy, A., & Hofmann, H. (2014). HLMdiag: A suite of diagnostics for hierarchical linear models in R. *Journal of Statistical Software*, *56*, 1–28.

Maas, C. J., & Hox, J. J. (2004). The influence of violations of assumptions on multilevel parameter estimates and their standard errors. *Computational Statistics & Data Analysis*, *46*(3), 427–440.
